# High‐Performance Electrocatalysts of Potassium Lactate Oxidation for Hydrogen and Solid Potassium Acetate Production

**DOI:** 10.1002/adma.202419578

**Published:** 2025-02-13

**Authors:** Jun Hu, Xintong Gao, Shanqing Li, Zhongsheng Xie, Xinyu Sheng, Zhixiang Yuan, Fei Zhang, Ping Chen, Yao Zheng, Shi‐Zhang Qiao

**Affiliations:** ^1^ School of Materials Science and Engineering Anhui University Hefei 230601 P. R. China; ^2^ School of Chemical Engineering The University of Adelaide Adelaide SA 5005 Australia; ^3^ Anhui Engineering Research Center of Highly Reactive Micro‐Nano Powders Chizhou University Chizhou 247000 P. R. China

**Keywords:** electrocatalysts, hydrogen, plastic waste, polylactic acid, potassium acetate

## Abstract

With the increasing use of polylactic acid (PLA), more attention is turning to its post‐treatment. Current methods such as natural decomposition, composting, and incineration are limited by significant carbon dioxide emissions and resource waste. Here, an efficient electrocatalytic conversion approach is presented to transform PLA waste into high‐value chemicals, particularly potassium acetate (AA‐K). By combining experimental and theoretical calculation, a high‐performance catalyst Ni(Co)OOH is developed, which exhibits a current density of 403 mA cm⁻^2^ at 1.40 V (vs RHE) with 96% Faraday efficiency for AA‐K in the electrooxidation of potassium lactate (LA‐K, the product of PLA degradation in KOH). Through in situ spectroscopy techniques and density functional theory calculations, the structural regulation of the catalyst, and reaction pathways of the electrooxidation are elucidated. Further experiments demonstrate the superior catalytic performance of the Ni(Co)OOH catalyst in an industrial‐scale tandem system. In 2 h of electrolysis, 320 g of PLA waste produces 232 L of H₂, yielding 1200 g of AA‐K with 97% purity after neutralization and drying. The system demonstrates high conversion efficiency (approaching 97%) for diverse real PLA waste forms, including powder, cups, fibers, and cloth. This research provides a scalable and sustainable approach for PLA waste upcycling.

## Graphical abstract

Potassium lactate, produced from polylactic acid plastic waste, is oxidized to potassium acetate on an anode using Ni(Co)OOH as the electrocatalyst, while hydrogen is simultaneously generated at the cathode, through a highly efficient industrial‐scale electrolysis system.

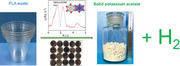

## 1 Introduction

Polylactic acid (PLA) is a representative renewable polymer material that plays a pivotal role in the chemical industry.^[^ ^1^ ^]^ However, increasing attention is being directed toward the post‐treatment of PLA waste.^[^ ^2^ ^]^ Current treatment methods include natural decomposition, composting, and incineration, which generate significant amounts of carbon dioxide; contributing to carbon emission and resources waste.^[^ ^3^ ^]^ Therefore, developing innovative strategies for treating PLA waste while simultaneously producing high‐value‐added products is of substantial importance for environmental protection and resource utilization.^[^ ^4^ ^]^

Electrocatalytic oxidation of small molecules represents a promising approach by enabling hydrogen (H₂) production at the cathode while generating high‐value‐added products at the anode.^[^ ^5^ ^]^ The degradation of PLA waste into lactic acid (LA), rather than aldehydes or other byproducts, is particularly desirable due to LA's relative stability and reduced susceptibility to non‐Faraday side reactions during subsequent electrochemical oxidation processes.^[^ ^1c^ ^]^ Theoretically, the electrooxidation of LA to acetate (AA) has a lower equilibrium potential (e.g., 0.2–0.3 V vs RHE) compared to water oxidation.^[^ ^6^ ^]^ Moreover, in an alkaline electrolyte, PLA is initially degraded to potassium lactate (LA‐K), which undergoes electrooxidation to form high‐value potassium acetate (AA‐K) while generating H_2_ at the cathode. However, research on the electrooxidation of LA‐K (LOR) is still in its infancy. Current studies are primarily confined to three‐electrode systems, with a maximum electrolyte volume of 50 mL and the best‐reported reaction performance achieved at ≈50 mA cm⁻^2^, 1.4 V (vs RHE).^[^ ^6^ ^]^ Additionally, the lack of comprehensive investigations into the reaction mechanisms has impeded further advancement in this field.

Recent advancements in electrocatalytic oxidation of small molecules, such as methanol, ethanol, glycerol, and urea, coupled with H_2_ production, have shown remarkable progress. However, these reactions have not yet been implemented at an industrial scale due to two primary challenges: ^[^ ^5^, ^7^ ^]^ (1) insufficient selectivity of the target products and (2) strong alkaline environment of these reactions. The latter results in alkali‐rich electrolytes, making product separation and purification challenging, thereby limiting the production of high‐purity target products.^[^ ^8^ ^]^ In the case of PLA waste, the conversion of PLA into high‐purity AA‐K while generating H_2_ in an alkaline environment holds significant importance. However, implementing this process on an industrial scale presents considerable challenges. The electrolysis process is crucial for successful implementation; it requires an anode electrocatalyst that can achieve high current density, selectivity, and large‐scale preparation to produce pure AA‐K efficiently. Additionally, subsequent treatment of the electrolyte containing KOH must not only guarantee the high purity of the final product AA‐K but also ensure that the treatment process incurs minimal economic costs.

Here, we have successfully developed an industrial‐scale tandem system that integrates PLA pretreatment, electrolysis, and post‐treatment processes to convert PLA waste into pure solid AA‐K while simultaneously generating H_2_. In a cyclic process of the tandem system, 320 g of PLA waste is initially treated with a KOH solution. Following two h of electrolysis, this process produces 232 L of H_2_. Subsequently, the reaction electrolyte is neutralized using AA to adjust the pH value. The final step involves spray drying, resulting in a product yield of 1200 g of pure AA‐K. The core component of this system is an industrial electrolyzer with an area of 1386 cm^2^ for both anode and cathode. The high‐performance anode catalyst Ni(Co)OOH, which has been developed through a combination of experimental and theoretical approaches, plays a crucial role in the operation of the system. The catalyst exhibits a current density of 403 mA cm⁻^2^ at 1.40 V (vs RHE) with 96% Faraday efficiency for AA‐K production (FE_AA‐K_) during LOR. Through in situ spectroscopy techniques, including infrared spectroscopy and X‐ray absorption fine structure (XAFS), combined with density functional theory (DFT) calculations, we elucidate the evolution of valence states, mechanisms of structural regulation, and reaction pathways of the electrocatalyst during LOR. An economic analysis indicates that this system possesses significant economic value, thereby validating its feasibility for practical application.

## 2 Results and Discussion

**Figure 1:**
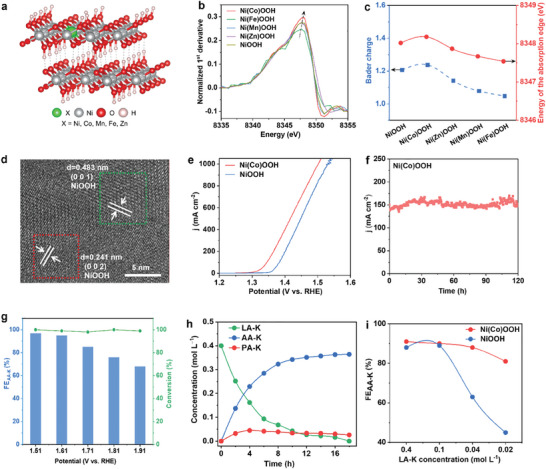
Catalyst structure prediction and electrocatalytic performance for LOR. a) Geometric structures of NiOOH, Ni(Co)OOH, Ni(Mn)OOH, Ni(Fe)OOH, and Ni(Zn)OOH. b) Normalized first‐derivative *K*‐edge spectra of the aforementioned catalysts. c) Relationship between Bader charge and energy of the absorption edge. d) HRTEM image of Ni(Co)OOH. e) Electrocatalytic performance comparison of Ni(Co)OOH and NiOOH catalysts for LOR. f) *I–t* curve of Ni(Co)OOH catalyst (0.5 cm^2^ of geometric area) at 1.51 V (vs RHE, without iR correction). Since LA‐K is consumed during the LOR process, we refresh the electrolyte. g) FE_AA‐K_ and LA‐K conversion for Ni(Co)OOH catalyst at different potentials (without *iR* correction). h) Concentration of LA‐K, AA‐K, and PA‐K as a function of time during a reaction cycle. i) Relationship between FE_AA‐K_ and initial concentration of LA‐K for Ni(Co)OOH and NiOOH.

Oxide‐derived NiOOH is regarded as the active component of catalysts in small molecules (methanol, hydrazine, urea, amines, etc.) electrooxidation.^[^ ^9^ ^]^ Enhancing the catalytic performance of NiOOH through doping with transition metal elements has emerged as an effective modification strategy to enhance its selectivity and activity for target products.^[^ ^10^ ^]^ Initially, we selected four elements (Co, Mn, Fe, and Zn) as potential dopants to replace Ni sites in NiOOH (**Figure** **1**a). Bader charge analysis reveals that the electronic structure of NiOOH is significantly influenced by doping, with the charge distribution following the order: Ni(Co)OOH > NiOOH > Ni(Zn)OOH > Ni(Mn)OOH > Ni(Fe)OOH. To validate these theoretical insights, we synthesized various doped catalysts and characterized them using X‐ray absorption spectroscopy (XAS). The results indicate that doping, particularly with Co, increases the oxidation state of Ni (Figure 1b),^[^ ^11^ ^]^ which is consistent with our DFT calculation results (Figure 1c). High‐resolution transmission electron microscopy (HRTEM) images (Figure 1d) and mapping images of Ni, Co, and O elements (Figure S1, Supporting Information) indicate that the main component of the Ni(Co)OOH catalyst is Co‐doped NiOOH. Ni(Co)OOH catalyst is derived from Ni_3_P_2_O_8_^.^8H_2_O with Co element supported on the nickel foam (designated as NiCo‐PO, Figure S2, Supporting Information). Normalized XANES spectra of Fe *K*‐edge, Mn *K*‐edge, and Zn *K*‐edge indicate that the elements Fe, Mn, and Zn are successfully doped into Ni(Fe)OOH, Ni(Mn)OOH, and Ni(Zn)OOH, respectively (Figure S3, Supporting Information).

LOR performance for Ni(Co)OOH and NiOOH catalysts was tested in 1 mol L^−1^ KOH with 0.4 mol L^−1^ LA‐K solution via a typical three‐electrode system. At a potential of 1.40 V (vs RHE), Ni(Co)OOH can achieve a current density of 403 mA cm^−2^, which is greater than that of NiOOH (210 mA cm^−2^) and that of CoOOH (187 mA cm^−2^) (Figure 1e; Figures S4 and S5a, Supporting Information). From Figure S5b (Supporting Information), it is evident that the molar ratio of Ni to Co in the catalyst has a significant impact on its catalytic activity. Compared to other elements, co‐doping achieves the highest current density (Figure S5c, Supporting Information).

Notably, Ni(Co)OOH exhibits superior catalytic activity compared to all previously reported LOR catalysts.^[^ ^6^ ^]^ Stability testing for LOR over 125 h confirms the exceptional durability of Ni(Co)OOH (Figure 1f). More importantly, Ni(Co)OOH demonstrates high LA‐K conversion and AA‐K productivity. As is shown in Figure 1g and Figures S5d and S6 (Supporting Information), Ni(Co)OOH exhibits a remarkable 98% conversion of LA‐K and a 96% FE_AA‐K_ (other doped NiOOH catalysts have similar FE_AA‐K_) at 1.61 V. In addition, Ni(Co)OOH converts LA‐K to AA‐K in each cycle with less than 4% by‐product potassium pyruvate (PA‐K) (Figure 1h). At a reduced LA‐K concentration of 0.02 mol L^−1^, Ni(Co)OOH maintained an FE_AA‐K_ of 81%, significantly outperforming NiOOH (FE_AA‐K_ = 45%) (Figure 1i; Figure S7, Supporting Information).

In situ Raman spectroscopy provides direct evidence for the formation of NiOOH during LOR. For NiCo‐PO, a pair of Raman signals at 474 cm^−1^ and 556 cm^−1^ emerges when the applied voltage reaches 1.35 V (vs RHE), with their intensity increasing as the potential rises, indicating the formation of NiOOH (Figure S8a, Supporting Information).^[^ ^12^ ^]^ In contrast, for Ni‐PO, a similar pair of Raman signals appear at 474 and 556 cm^−1^ when the applied voltage reaches 1.40 V (vs RHE), also exhibiting enhanced intensity with increasing potential (Figure S8b, Supporting Information). Compared to Ni‐PO, NiCo‐PO demonstrates a greater propensity for producing NiOOH.

**Figure 2:**
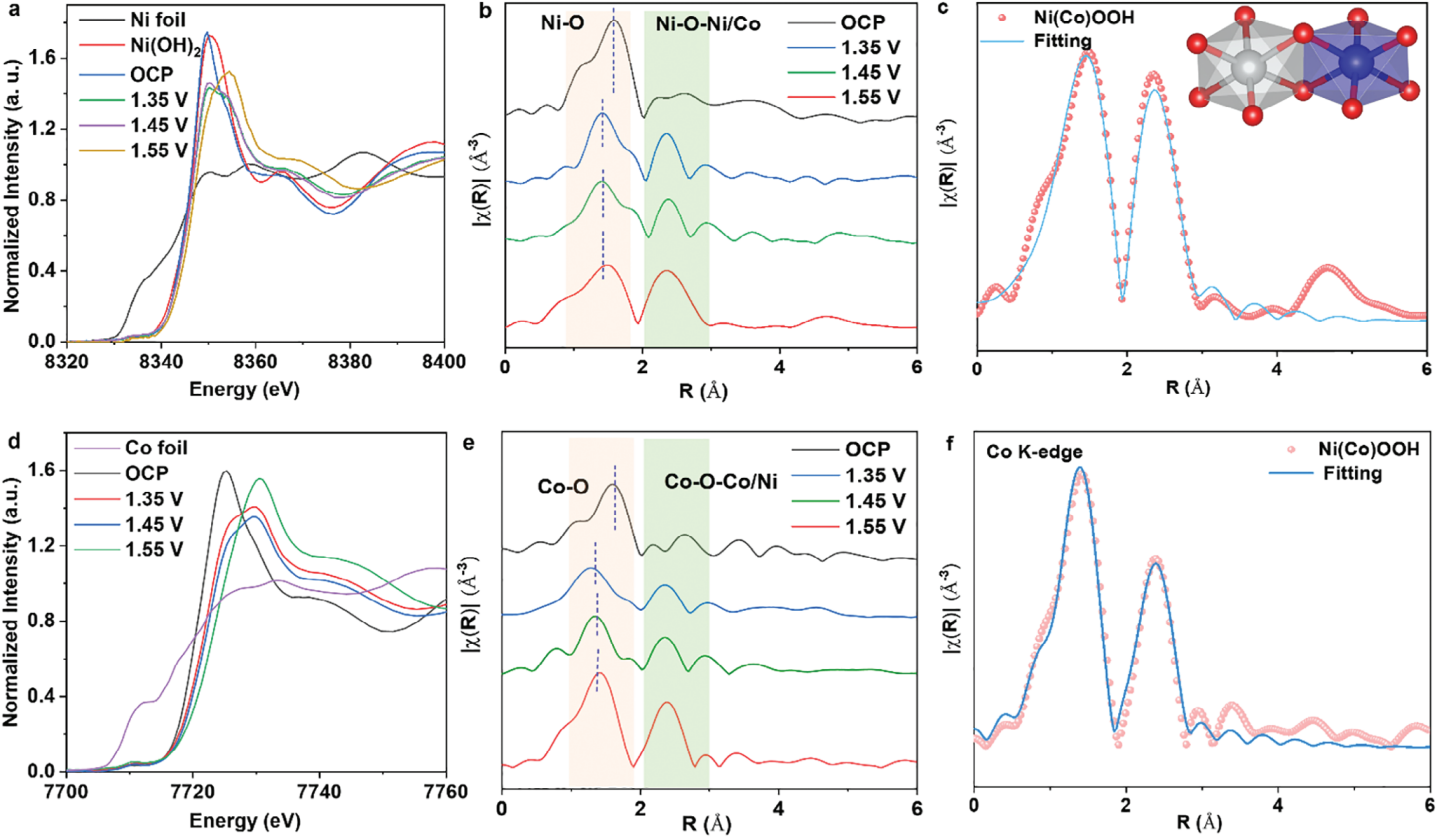
In situ XAFS measurements. a) Normalized in situ XANES spectra of Ni *K*‐edge for the pre‐catalyst for Ni(Co)OOH at different potentials. b) In situ FT‐EXAFS spectra of Ni *K*‐edge for pre‐catalyst at different potentials. c) The fitting curves of *k* ^2^‐weighted FT‐EXAFS spectra of Ni *K*‐edge for Ni (Co)OOH. d) Normalized in situ XANES spectra and e) In situ FT‐EXAFS spectra of Co *K*‐edge for pre‐catalyst for Ni(Co)OOH at different potentials. f) The fitting curves of *k* ^2^‐weighted FT‐EXAFS spectra of Co *K*‐edge for Ni(Co)OOH.

In situ, X‐ray absorption near edge structure (XANES) and extended X‐ray absorption fine structure (EXAFS) analyses were used to monitor the real‐time evolution of the local metal center structure in Ni(Co)OOH catalyst during LOR. As shown in **Figure** **2**a, the Ni *K‐*edge XANES spectra exhibit a positive shift in the absorption edge with increasing applied potential, indicating an increase in the average oxidation state of Ni during LOR. At 1.35 V, the first‐shell Ni‐O bond length decreases from 1.62 to 1.38 Å, while the second‐shell double peak transforms into a single peak with increased intensity, suggesting a reconfiguration of the Ni coordination environment (Figure 2b). This structural rearrangement generates low‐symmetry and unsaturated Ni sites with structural distortions. At 1.45 V, both the first and second shell bond lengths remain consistent with those at 1.35 V, but their peak intensities increase significantly, indicating the formation of highly symmetric NiO_6_ octahedra (β‐NiOOH).^[^ ^13^ ^]^ At 1.55 V, the peak intensities of Ni‐O and Ni─O─Ni/Co reach their maximum, demonstrating the formation of a fully coordinated β‐NiOOH structure. Quantitative structural parameters obtained from *K*‐edge FT‐EXAFS data of Ni in Ni(Co)OOH indicate that at 1.55 V, the Ni‐O bond length in the first shell is 1.93 Å with a coordination number of 5.92, while the Ni─O─Ni/Co bond length in the second shell is 2.85 Å with a coordination number of 5.98 (Figure 2c; Table S1, Supporting Information).^[^ ^14^ ^]^ These findings suggest that the coordination environment of Ni in Ni(Co)OOH is consistent with that in NiOOH. Additionally, wavelet transform analysis of Ni *K*‐edge shows maximum dispersion centers at 4.4 and 6.1 Å⁻¹, corresponding to Ni─O and Ni─O─Ni/Co bonds, respectively. Notably, during the transition from open circuit potential (OCP) to 1.35 V, the second‐shell dispersion center gradually emerges, indicating the structural transformation of precatalyst from its initial state to a hydroxide‐like phase. This center intensifies with increasing potential, culminating in the formation of a highly ordered β‐NiOOH structure (Figure S9, Supporting Information).

The structural evolution of Co centers during LOR was also tracked. As shown in Figure 2d, the Co *K*‐edge absorption edge exhibits a positive shift with increasing potential, similar to Ni, indicating that Co also participated in the catalytic process as active sites. Co EXAFS data reveals that the evolution of the Co coordination environment (in terms of bond lengths and coordination numbers) is similar to that of the Ni coordination environment with increasing potential (Figure 2e,f; Tables S2, Supporting Information), highlighting the cooperative role of Ni and Co as active centers in Ni(Co)OOH structure to drive the catalytic reaction.^[^ ^15^ ^]^

**Figure 3:**
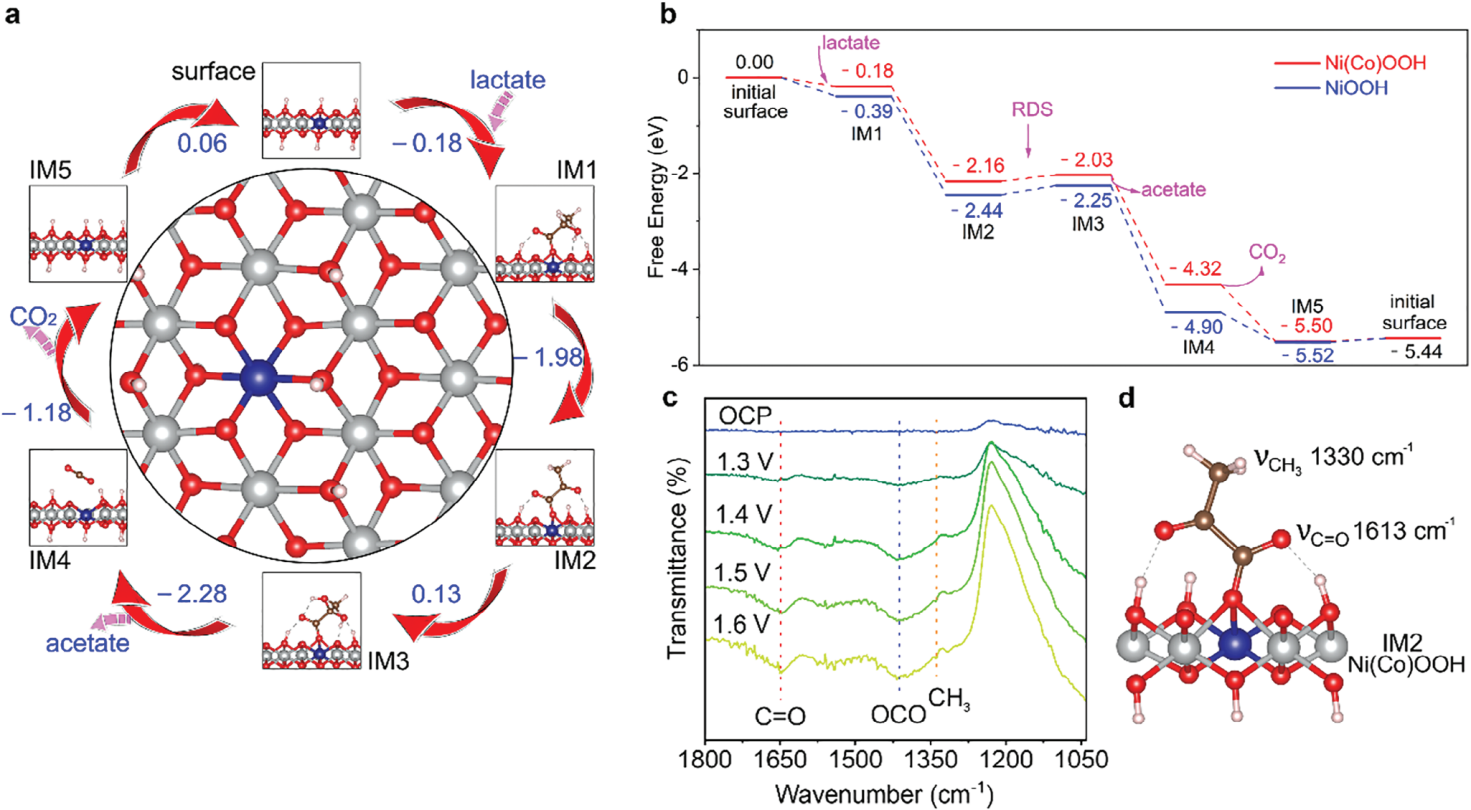
Mechanism for LOR. a) Geometric structures of the Ni(Co)OOH catalyst and corresponding reaction intermediates. b) Reaction pathway of LA‐K oxidation and Gibbs free energy changes at 1.32 V on NiOOH and Ni(Co)OOH catalysts. c) ATR‐SEIRAS during LOR. d) The simulated results of harmonic vibrational frequencies in LOR pathway.

The reaction pathway for LOR catalyzed by Ni(Co)OOH and NiOOH was proposed in **Figure** **3**a,b. The Bader charge of the neighboring Ni atom increases from +1.414 to +1.461e, the Ni‐O (H) bond length decreases from 1.409 to 1.402 Å, and the Ni─O─Ni bond length changes from 1.889+1.996 to 1.873+1.946 Å. These minor changes do not significantly alter the catalytic reaction pathway or mechanism but affect the energy of the elementary reaction. In the rate‐determining step (RDS) of the LOR (IM2+OH^−^→IM3+e^−^), Ni(Co)OOH catalyst exhibits a lower Gibbs free energy (0.13 eV) compared to NiOOH (0.19 eV), indicating that Ni(Co)OOH's superiority (Tables S3–S5, Supporting Information). In addition, for the competitive OER reaction, Ni(Co)OOH exhibits a higher energy barrier (*O+OH^−^→*OOH+e^−^, 0.49 eV) than NiOOH (0.45 eV), demonstrating its enhanced ability to suppress oxygen production (Figure S10, Supporting Information).

The previous discussion indicates that the molar ratio of Ni to Co in catalysts significantly affects catalytic performance (Figure S5b, Supporting Information). DFT results reveal the synergistic interaction between Ni and Co in catalytic reactions. Specifically, both Ni and Co serve as active sites in the catalytic process, actively participating in electron transfer. In the presence of Co, Ni loses more electrons, resulting in an increased oxidation state of Ni.^[^ ^16^ ^]^ This phenomenon is consistent with the X‐ray absorption near‐edge structure (XANES) experiment, where the normalized first derivative of the *K*‐edge spectra supports that Co not only participates in electron transfer but also modulates Ni's electronic structure by “plundering” electrons from Ni. It is evident that the synergistic effect between Ni and Co is a key factor in enhancing catalytic performance. For the optimal Ni₀.₈Co₀.₂OOH catalyst, with a Ni to Co ratio of 8:2, the interaction between Ni and Co reaches its peak, effectively utilizing Ni's high catalytic activity while Co further optimizes Ni's electronic structure, thereby enhancing the catalytic performance. The subtle electronic interactions between Ni and Co, especially the “plundering” effect of Co on Ni's electron density, are crucial for improving catalytic efficiency.^[^ ^17^ ^]^

In situ, attenuated total reflectance surface‐enhanced infrared absorption spectroscopy (ATR‐SEIRAS) analysis was performed to identify the critical reaction intermediates predicted by DFT calculation (Figure 3c). The bands observed at 1650, 1415, and 1340 cm⁻¹ correspond to the stretching vibration of C═O, the stretching vibration of OCO, and the deformation vibration of CH_3_ associated with carboxylic acid adsorption, respectively. These bands were detected under LOR conditions and exhibited increased intensity with increasing potential.^[^ ^18^ ^]^ These findings align with simulated results of harmonic vibrational frequencies, corroborating the formation of intermediates during LOR (Figure 3d).

To evaluate PLA electrochemical upgrading, we constructed a commercial flow membrane electrode assembly (MEA) electrolyzer with Ni(Co)OOH catalyst as the anode and Pt/Ti fiber felt as the cathode (working areas of 4 cm^2^ each) (Figure S11a, Supporting Information).^[^ ^19^ ^]^ 1 mol L^−1^ KOH solution with 0.4 mol L^−1^ LA‐K was used as the anode electrolyte and 1 mol L^−1^ KOH solution was used as the cathode electrolyte. LA‐K is converted into AA‐K within 3 h at a cell voltage of 2.0 V with a conversion efficiency exceeding 99.0%, and the yield and FE_AA‐K_ as high as 0.91 g cm^−2^ and 91.2%, respectively (Figure S11b–d, Supporting Information). In addition, the cathode H_2_ production reaches a high level of 0.41 L cm^−2^. The electricity inputs for LA‐K upcycling are only 8.86 Wh per cycle, which demonstrates excellent energy‐saving efficiency (Figure S11e, Supporting Information). In 35 consecutive cycles, the concentration of product AA‐K remained stable at ≈0.38 mol L⁻¹, while the FE_AA‐K_ consistently approached 96%. These findings indicate that the Ni(Co)OOH catalyst demonstrated remarkable stability during the electrolysis process (Figure S11f, Supporting Information). The superior performance of Ni(Co)OOH catalyst in MEA provides a basis for industrial applications.

**Figure 4:**
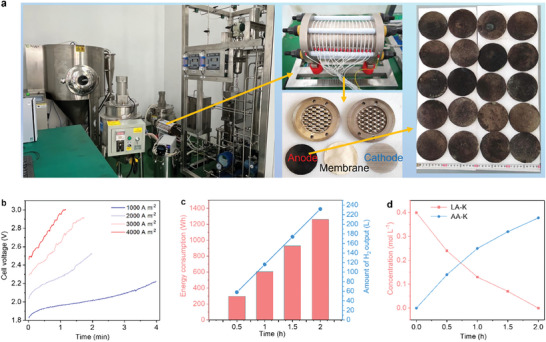
Performance of the industrial‐scale electrolysis device. a) Photographs of the electrolyzer, each chamber, and Ni(Co)OOH catalyst. b) Relationship between cell voltage and time at different current densities during a cycle (10 L electrolyte). c) Energy consumption and H_2_ output over time in a cycle. d) Concentration of LA‐K and AA‐K over time in a cycle.

Based on the exceptional performance of the MEA electrolyzer, the Ni(Co)OOH catalyst was employed as the anode, and commercially available nickel wire mesh was used as the cathode to develop an industrial‐scale electrolysis device. This device consists of an alkaline water electrolyzer, a gas‐liquid separator, and a heating and temperature control system. The electrolyzer serves as the core component of the device. It comprises 18 individual electrolytic cells, with a total electrolyte volume of 10.0 L. Additionally, the working area for both the anode and cathode measures 1386 cm^2^ (**Figure** **4**a). In this industrial‐scale electrolysis, LA‐K derived from PLA degradation in KOH is subsequently oxidized to AA‐K at the anode while generating H₂ at the cathode. After a 2 h cycle at a current density of 2000 A m^−2^, 363.8 g of AA‐K and 232 L of H_2_ are produced with 1.26 KWh energy consumption (Figure 4b,c). From Figure 4d, the concentration of LA‐K gradually decreases in the electrolysis, while the concentration of the target product AA‐K gradually increases.

**Figure 5:**
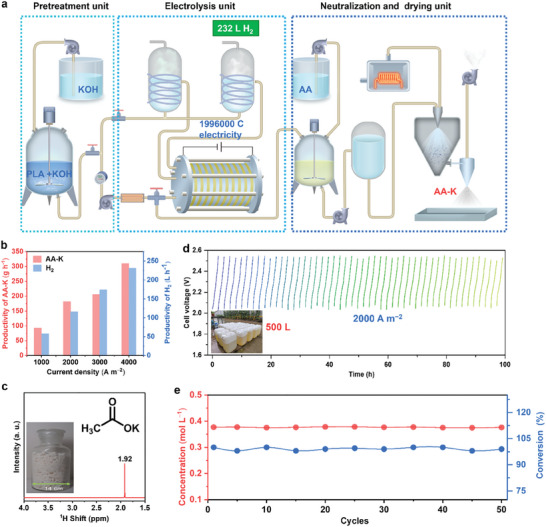
A tandem industrial‐grade system for pure H_2_ and solid AA‐K. a) Process flowchart for the system. b) Productivity curves of AA‐K and H_2_ as a function of current density during a reaction cycle. c) ^1^H NMR spectra of the final product AA‐K. Inset, photo of the final product from a reaction cycle. d) Stability evaluation of the electrochemical process under a constant current density (2000 A m^−2^) at 70 °C. Inset, photo of the electrolyte produced by a total of 50 electrolytic cycles (10 L per cycle). e) Concentrations of AA‐K and the conversion of LA‐K during 50 consecutive cycles.

Due to the high efficiency of the industrial‐grade device, we also have established an industrial‐scale electrosynthesis system for extracting AA‐K and H_2_ from PLA (**Figure** **5**a). The system primarily comprises an alkali treatment device (reaction tank), an electrolysis device, an acid treatment device (reaction tank), and a drying apparatus (spray dryer). The specific operation is as follows: PLA is first treated with KOH solution to obtain LA‐K solution, which is then oxidized to AA‐K at the anode while generating H_2_ at the cathode in the industrial‐scale electrolyzer. After acid treatment and drying, solid AA‐K is obtained. In the system's cycle process (at a current density of 2000 A m^−2^), 320 g of PLA waste is treated with KOH solution and electrolyzed for 2 h, yielding 232 L of H_2_ (Figure 5b). The pH of the electrolyte is subsequently adjusted and the final electrolyte undergoes a spray‐drying process to yield 1200 g of pure solid AA‐K. The resulting AA‐K product exhibits over 97% purity, as confirmed by ^1^H NMR spectra, infrared spectra, and ion chromatography data (Figure 5c; Figure S12a,b, Supporting Information). In addition, the Ni(Co)OOH catalyst anode demonstrates ultra‐high stability, maintaining over 50 cycles (100 h) at 2000 A m^−2^. In this process, the system processes 500 L of LA‐K solution, producing 11.6 Nm^3^ of H_2_, achieving industrial‐grade scale (Figure 5d,e; Figure S12c,d, Supporting Information). Excitingly, to the best of our knowledge, this is the first reported industrial‐scale LA‐K electrooxidation system.

**Figure 6:**
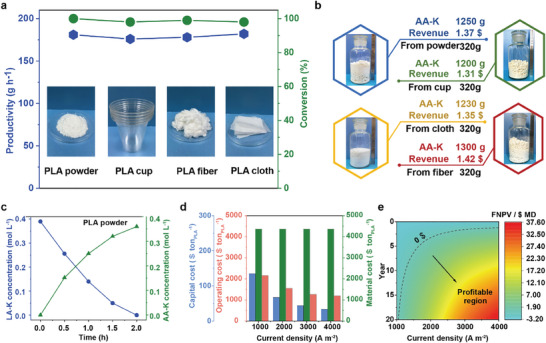
Implementation of PLA waste to solid AA‐K and H_2_ by the proposed tandem industrial‐grade system. a) Productivity of AA‐K and conversion of LA‐K during the electrolysis of waste PLA powder, PLA cup, PLA fiber, and PLA cloth during a reaction cycle. b) Quantities and images of solid AA‐K derived from various actual PLA waste sources. c) Concentration curves of LA‐K and AA‐K as a function of time during an electrolytic cycle. d,e) Economic profitability and cost analysis of the industrial‐grade system for converting PLA waste into H_2_ and AA‐K products.

With the increasing accumulation of PLA waste, effective recycling is crucial for mitigating environmental pollution and recovering economic value.^[^ ^4^, ^20^ ^]^ Diverse real PLA wastes, including powders, cups, fibers, and cloths, are converted through the system, and high conversion efficiencies (approaching 97%) can be achieved (**Figure** **6**a,b). For instance, concentration curves of LA‐K and AA‐K within the LA‐K solution derived from 320 g of PLA powder during 2‐hour electrolysis further substantiate that discarded PLA can achieve a remarkably high conversion rate (Figure 6c). To evaluate the economic feasibility of the system, a preliminary techno‐economic analysis (TEA) was conducted.^[^ ^21^ ^]^ We considered all costs associated with industrial‐scale electrolysis and subsequent chemical processes, including electrolyzer costs, ancillary equipment and plant infrastructure investment, electricity and feedstock consumption (PLA waste, KOH, and AA), labor and maintenance costs, etc. As is shown in Figure 6d, converting 1 ton of PLA waste into AA‐K results in a net income of $542, indicating the system's excellent economic potential. Furthermore, we analyzed the initial capital expenditure and operating cost over a 20‐year period using financial net present value (FNPV) to determine profitability. As is shown in Figure 6e, FNPV increases with current density and years of operation. At a current density of 2000 A m^−2^, the system generates an annual profit of $1044954, achieving investment recovery by the fourth year of operation, with an FNPV of $12.4 million by the end of its operational lifespan.^[^ ^22^ ^]^

## 3 Conclusion

In this study, we developed a high‐performance LOR electrocatalyst (NiCoOOH) that exhibits excellent activity and remarkable stability. Through in situ spectroscopy techniques, including infrared and XAFS, alongside density functional theory (DFT) calculations, we elucidated the valence evolution, structural regulation mechanisms, and reaction pathways of Ni(Co)OOH catalyst during LOR reaction. Furthermore, we clarify the synergistic role of Ni and Co active species and highlight the contributions of key intermediates to the catalytic efficiency. The electrocatalyst was assembled into an industrial alkaline electrolyzer as the anode (1386 cm^2^) in an industrial‐scale PLA upgrading system, which contains KOH treatment of PLA waste, industrial‐grade electrolysis, AA treatment, and spray drying. Under operational conditions (2000 A m^−2^), this system achieved 98.0% conversion of LA‐K, yielding 232 L of H₂ per electrolysis cycle. Subsequent treatment produced 1200 g of solid AA‐K with a purity of exceeding 97%. The system demonstrates compatibility with various forms of PLA waste, including powders, cups, fibers, and cloths, maintaining high conversion. This study provides a scalable and sustainable approach for recycling waste plastics while delivering high‐value products.

## 4 Experimental Section

### Preparation

Ni(Co)OOH catalyst was prepared as follows: First, nickel foam (NF) was activated by sequential treatment with 1 m HNO_3_, absolute ethanol, and deionized water. Then, nickel chloride hexahydrate, cobalt chloride hexahydrate, and diamine hydrogen phosphate were dissolved in deionized water in a molar ratio of 8:2:12. The activated nickel foam was added to this solution in a beaker and heated at 70 °C for 3 h. After cooling to room temperature, the product was collected, washed with ethanol and deionized water, and dried at 40 °C for 6 h. Ni(Co)OOH catalyst was derived from the above product by an electrochemical oxidation in 1.0 mol L^−1^ KOH. NiOOH catalyst was prepared using the same as Ni(Co)OOH catalyst, except cobalt chloride hexahydrate was excluded. The preparation of Ni(Zn)OOH, Ni(Mn)OOH, and Ni(Fe)OOH catalysts follows a procedure similar to that of NiOOH. However, it necessitates the incorporation of zinc chloride (1.09 g), manganese chloride (1.00 g), and ferrous chloride (1.01 g), respectively.

### Characterization

Transmission electron microscopy (TEM) images were conducted on JEM‐F200 (Japan) instrument. Inductively Coupled Plasma Optical Emission Spectrometry (ICP‐OES) was performed using EXPEC6100 (China).

### Electrochemical Measurements

Linear sweep voltammetry (LSV), electrochemical impedance spectroscopy (EIS), and chronopotentiometry (CP) were performed by an IM6e electrochemical workstation (Zahner‐Electrik, Germany) in a divided H‐type electrochemical cell. The LSV curves were obtained at a scan rate of 5 mV s^−1^. The electrocatalysts (NiCoOOH or NiOOH, 1.0 cm^2^) were directly used as the working electrode, with Hg/HgO as the reference electrode and Pt mesh as the counter electrode. The anode and cathode compartments were separated by an anion exchange membrane (FAA‐3‐PK‐130) and contained anolyte (80 mL of 1 mol L^−1^ KOH with 0.4 mol L^−1^ LA‐K) and catholyte (80 mL of 1 mol L^−1^ KOH). All curves are manually corrected with iR compensation (except for special instructions). A flow MEA with a working area of 4 cm^2^ was used to evaluate the catalyst performance. Ni(Co)OOH served as the anode and Pt/Ti fiber felt served as the cathode. The anode electrolyte (80 mL of 1.0 mol L^−1^ KOH with 0.4 mol L^−1^ LA‐K) and the cathode electrolyte (80 mL of 1.0 mol L^−1^ KOH) were circulated using a peristaltic pump at a flow rate of 40 mL min^−1^.

### Measurement of Reactants And Products

The concentrations of LA‐K, AA‐K, and PA‐K were analyzed using an IC6210 ion chromatography system (IC, Wayeal AS2800, China), which is equipped with a HS‐5A‐P3 anion‐exchange column (4.0 × 250 mm).^[^ ^23^ ^]^ Each measurement was performed three times and the average value was calculated.

### In Situ Measurements

The X‐ray absorption spectroscopy (XAS) data were collected in fluorescence mode at the BL14W1 beamline of the Shanghai Synchrotron Radiation Facility. The storage ring operated at an energy of 3.5 GeV with a maximum current of 250 mA. The working electrode consisted of a carbon cloth loaded with the catalyst, a platinum wire was used as the counter electrode, and an Ag/AgCl electrode served as the reference.^[^ ^14^ ^]^ In situ XAS measurements were conducted in a 1 mol L^−1^ KOH solution containing LA‐K as the electrolyte. The X‐ray absorption near‐edge structure (XANES) data were normalized using the ATHENA module of the Demeter software package. In situ ATR‐SEIRAS tests were conducted at Hefei in Situ Technology Co., LTD. The Fourier transform infrared spectrometer (FTIR, Nicolet iS50) consists of an electrochemical reaction cell with a silicon window and a liquid nitrogen‐cooled MCT detector.^[^ ^24^ ^]^ The electrochemical spectra were acquired by accumulating 32 scans at a resolution of 4 cm⁻¹, during which a stable potential was applied to the working electrode by an electrochemical workstation. The electrolyte solution comprised 1.0 mol L^−1^ KOH with 0.4 mol L^−1^ LA‐K. For the preparation of the working electrode, 5.0 mg of catalyst and 10 mL of Nafion solution (5 wt.%) were dispersed in 590 µL of isopropanol through sonication for 30 min to create a homogeneous ink. Subsequently, 10 µL of this uniform ink was applied onto singular silicon crystals that had been previously coated with a layer of Au and allowed to dry under ambient conditions. In situ Raman measurements were conducted using a Via‐Reflex spectrometer (Renishaw) with a laser excitation wavelength of 532 nm. The measured potential ranged from 1.2 to 1.6 V. The in situ electrochemical three‐electrode cell comprised a Ni(Co)OOH electrocatalyst as the working electrode, Ag/AgCl as the reference electrode, and a Pt wire as the counter electrode.

### Performance Evaluation of the Tandem Industrial‐Grade System

This system mainly contains a base treatment device, an electrolysis device, an acid treatment device, and a spray dryer. The electrolysis device was bought from SuZhou Moor Gas Equipment Co. Ltd. The electrolyzers consist of 18 cells with a total electrolyte volume of 10.0 L and an electrolytic area of 1386 cm^2^ for both the anode and cathode. Ni(Co)OOH and commercial Ni wire mesh electrodes were used as the anode and cathode, respectively. The electrolysis was carried out in 10.0 L of 1.0 mol L^−1^ KOH solution and 0.4 mol L^−1^ LA‐K at 70 °C. After electrolysis, the concentrations of LA‐K, AA‐K, and PA‐K were tested. Product purity was analyzed using a nuclear magnetic resonance spectrometer (JNM‐ECZR600) and Fourier transform infrared spectrometer.

Details of DFT calculations and economic analysis are provided in the supporting information.

## Conflict of Interest

The authors declare no conflict of interest.

## Supporting information



Supporting Information

## Data Availability

The data that support the findings of this study are available from the corresponding author upon reasonable request.
