# Supplementary material for: High‐Performance Electrocatalysts of Potassium Lactate Oxidation for Hydrogen and Solid Potassium Acetate Production
Source: Adv Mater. 2025 Feb 13;37(13):2419578. doi: 10.1002/adma.202419578 (PMC11962691; doi:10.1002/adma.202419578)
Supplement: Supplementary file 1 — Supporting Information [file ADMA-37-2419578-s001.docx]

**Supporting Information**

High-Performance Electrocatalysts of Potassium Lactate Oxidation for Hydrogen and Solid Potassium Acetate Production

Jun Hu, ^†^ Xintong Gao, ^†^ Shanqing Li, ^†^ Zhongsheng Xie, Xinyu Sheng, Zhixiang Yuan, Fei Zhang, Ping Chen,* Yao Zheng,* Shi-Zhang Qiao *

[a] J. Hu, Z. Xie, X. Sheng, Z. Yuan, F. Zhang, Prof. P. Chen

School of Materials Science and Engineering, Anhui University, Hefei, 230601, P. R. China

Email: chenping@ahu.edu.cn

[b] X. Gao, Prof. Y. Zheng, Prof. S. Qiao

School of Chemical Engineering, The University of Adelaide, Adelaide, SA 5005, Australia

Email: [yao.zheng01@adelaide.edu.au](mailto:yao.zheng01@adelaide.edu.au), [s.qiao@adelaide.edu.au](mailto:s.qiao@adelaide.edu.au)

[c] S. Li

Anhui Engineering Research Center of Highly Reactive Micro-Nano Powders, Chizhou University, Chizhou, 247000, P. R. China

^†^ These authors equally contributed to this work.

**Supplementary Methods**

**DFT calculations**

The catalyst models and heterogeneous reactions were simulated using the Vienna ab Initio simulation package (VASP). The Perdew−Burke−Ernzerhof (PBE) functional and the projector augmented-wave (PAW) formalism were applied to geometry optimizations of periodic models. The vdW-dispersion energy-correction term was the DFT-D3(BJ).^[1]^ The second-order derivatives of the total energy with respect to the position of the ions were obtained by using the finite differences approach. The cutoff energy for the plane wave basis was set to 500 eV. The convergence threshold of the electronic self-consistency was specified as 1.0×10^-7^ eV, and the total energy change threshold between two ionic relaxation steps was designated as less than 0.02 eV. The interaction between a solute and a solvent was implemented by the implicit solvents model using the VASPsol code.^[2]^ Gibbs free energy changes of reactions were calculated as follows.

$$\Delta G=\Delta E+\Delta ZPE+\Delta\int_{0}^{T} C_{p}dT-T\Delta S+\Delta G_{sol}+\Delta G_{pH}+neU$$

where *E* is single-point energy, ZPE means zero-point energy, $\Delta\int_{0}^{T} C_{p}dT$ and $-T\Delta S$ stand for the correction factors of enthalpy and entropy, $G_{sol}$ is the solvation energy, $\Delta G_{pH}$ denotes the effect of hydrogen ions concentration on the energy, $neU$ stands for the correction of working voltage.

**Economic analysis**

In order to study the feasibility of the economic cycle of waste polylactic acid, we construct a techno-economic model. By designing the right process parameters to produce and sell reaction products at market prices, we can add value to the economy of the entire process system.^[3, 4]^

Through the market inquiry, it is understood that the electrolytic equipment at this scale can be customized by the manufacturer, the estimated price is $695,000, and the price of the salt extraction evaporator and hydrolyzer is $1.22 million. The main maintenance cost of the electrolyzer comes from the price of the electrode (catalyst), the cost of the electrode in the device is estimated to be $55.6 m^-2^, and the service life is 3 months. The cost of recovering PLA is taken into account in the cost of raw materials, and the price of PLA scrap is $548 ton^-1^. In addition, the main raw material is potassium hydroxide, part of it is used for PLA hydrolysis, part of it is used as electrolyte, and the amount of potassium hydroxide is 3.3 times that of PLA.

The main cost of the electrolyzer is the cost of the electrode to carry out the reaction process, in the following reaction process, the electrode area required for this process is about 282.6 m^2^. The optimal production conditions are based on a current density of 4000 A m^-2^, corresponding to a voltage of 2.5 V. Correspondingly, the reaction current can be computed as follows:

$$\text{I=}\text{I}_{\text{d}}\text{×}\text{S}_{\text{r}}\text{=4000A }\text{m}^{\text{-2}}\text{×282.6}\text{m}^{\text{2}}\text{=1130400A}$$

Where I is the total circuit current (A), I_d_ is the current density through the electrodes (A m^-2^) and Sr is the total electrode area (m^2^).

The total power consumption of the system for a single cycle :

$$\text{W=U×I×}\text{t}/\text{1000}\text{=2.5V×1130400A×1h/1000=2826kW·h}$$

Where W is the total electrical energy consumption (kW·h), U is the voltage (V) corresponding to the current density, I is the reaction current (A), and t is the current output time (h).

The hydrogen yield of a single cycle was calculated as:

$$\text{V=}\frac{\text{22.4×I×t×FE}}{\text{n×F×1000}}\text{= 463.2}\text{Nm}^{\text{3}}$$

Where V is the standard volume yield of hydrogen (Nm^3^), I is the reaction current (A), t is the single cycle reaction time (s), FE is the Faraday efficiency of hydrogen (100%) and n is the number of electrons transferred during the reaction, F=96500C.

1. **Calculation of investment in fixed assets.** The majority of the expense of the electrolyser in this procedure goes towards the electrodes. The cost of the electrodes can be determined by the experimental phase of catalyst preparation, which estimates it to be $55.6 m^-2^. Based on the scale of hydrogen production and existing industrial scenarios, the cost of electrolysis equipment for the process would be approximately $0.8 million dollars during the first year of operation. Moreover, to purify the potassium acetate in the electrolyte for selling purposes, a salt extraction evaporator and hydrolyzer set priced at about $1.22 million dollars can be bought for conducting salt extraction operations on the electrolyte.

$$\text{Stack cost=}\text{Electrolyzer}\text{ }\text{cost+Salt}\text{ evaporator cost}\text{ }\text{+ Hydrolyzer cos}\text{t}\text{=}\text{\$1912640}$$

In general, the cost of industrial plant construction is 35% of fixed assets, while the cost of equipment is 65% of fixed assets.

$$\text{Plant cost=Stack cost×}\text{0.35}/\text{0.65}\text{=}\text{\$}\text{102950}$$

The above fixed assets are depreciated on an annual basis, the project has an operating life of 20 years, and the interest rate is 5%.

$$\text{Depreciation of fixed capital=}\frac{\text{(1912640 +102950)×5\%}}{\text{1-}\frac{\text{1}}{\left( \text{1+5\%} \right)^{\text{20}}}}\text{=}\text{\$}\text{161736}$$

In general, annual equipment maintenance costs are calculated at 2.5% of the cost for electrolysis equipment:

$$\text{Maintenance cost=}\text{\$1912640}\text{×2.5\%=}\text{\$}\text{47816}$$

Based on the average wages of domestic workers, which is $13900/year per person, the labour cost for running the system amounts to roughly $69500/year. This is due to the system's small size and highly automated equipment, as well as the fact that the site only requires a small number of workers to maintain it.

1. **Calculation of operating costs.** Its primary running expenses entail electricity consumption for operating the device, maintenance of the device, labour and raw materials. The consumption of electricity in the system is the largest operating cost, which is calculated according to the domestic industrial electricity price of $0.55 kWh^-1^. The power consumption mainly comes from electrolytic cell, salt extraction evaporator, auxiliary equipment and so on. Among them, the auxiliary equipment (hydrolysis electricity, lighting electricity, machine pump electricity) electricity consumption is estimated to be 50% of the maximum electricity consumption of the electrolytic cell. The equipment power consumption covers both the electrolysis and salt extraction processes. The salt extraction process mainly involves the use of electricity from steam generators. Information from the salt evaporator manufacturer reveals the stack's minimum power is 420 kW·h m^-3^, translating to 8400 kW·h for the salt extraction process's primary cycle. We have estimated the energy consumption of the other equipment in the plant to be approximately 50% of the maximum power consumption of the electrolyser, including hydrolyzer, heat exchanger, pump and illumination. This power consumption remains independent of the current changes in the electrolysis process, then the annual electricity consumption is：

$$\text{Electricity cost=8000×(2826×1.5+8400)kWh×}\text{\$}\text{0.055}\text{kWh}^{\text{-1}}\text{=}\text{\$5561160}$$

Consequently, the fixed assets and operating costs mentioned above are transformed into the consumption of single-ton PLA treatment per year.

$$\text{Cost=}\frac{\text{5561160}\text{+}\text{161736}\text{+}\text{47816}\text{+}\text{69500+62850}}{\text{20000×8000×0.0003}}\text{=}\text{\$}\text{1230 }\text{ton}_{\text{PLA}}^{-1}$$

1. **Calculation of raw materials cost or profit.** For each cycle, the cost of raw materials and profit from by-products will be determined by a fixed purchase price based on the current market price: the price of pure KOH is $0.85 kg^-1^, the price of acetic acid is $0.42 kg^-1^ and the price of hydrogen is $0.51 Nm^-3^. The potassium acetate obtained from the salt extraction can be priced at $1.1 kg^-1^ on the market.

$$\text{KOH cost=0.997kg×2000×\$0.85}\text{kg}^{-1}\text{=\$1695}$$

$$\text{C}\text{H}_{\text{3}}\text{COOH cost=0.72kg×2000×\$0.42}\text{kg}^{-1}\text{=\$608}$$

$$\text{PLA cost=0.3kg×2000×}\text{\$0.54kg}^{-1}\text{=\$324}$$

$$\text{Materials cost=}\frac{\text{1695+608+324}}{\text{2000×0.0003}}\text{=}\text{\$}\text{4378}\text{ ton}_{\text{PLA}}^{-1}$$

$$\text{C}\text{H}_{\text{3}}\text{COOK profit=1.57kg×2000×}\text{\$1.1kg}^{-1}\text{=\$3454}$$

$$\text{H}_{\text{2}}\text{ profit=463.2}\text{Nm}^{\text{3}}\text{×\$0.51}\text{Nm}^{\text{-3}}\text{=\$236}$$

$$\text{Profit=}\frac{\text{236+3454}}{\text{2000×0.0003}}\text{=\$6150 }\text{ton}_{\text{PLA}}^{-1}$$

With an input condition of a current density of 4000A m^-2^, a corresponding voltage of 2.5V and an operating life of 20 years, the net profit is $542 ton_PLA_^-1^.

In addition, the FNPV is analyzed, indicating that the scheme is feasible and the investment benefit is good.

$$\text{FNPV=}\sum_{\text{t=0}}^{\text{n}} \text{(Cl-CO)}_{\text{t}}\text{×}\text{(1+i)}^{\text{-t}}$$

Where Cl is the present value of future cash flow, CO is the present value of the original investment, i is the discount rate, and t is the duration.

The negative FNPV values indicate an economic non-profit manufacture. The postive FNPV starts in the second year, indicating a payback time of two years. Besides the payback duration, the total economic benefits after the 20 year lifespan are other important evaluation parameters, and the FNPV ends at $37.5 million over the 20 year lifespan at 4000 A m^-2^.

**Supplementary Figures**


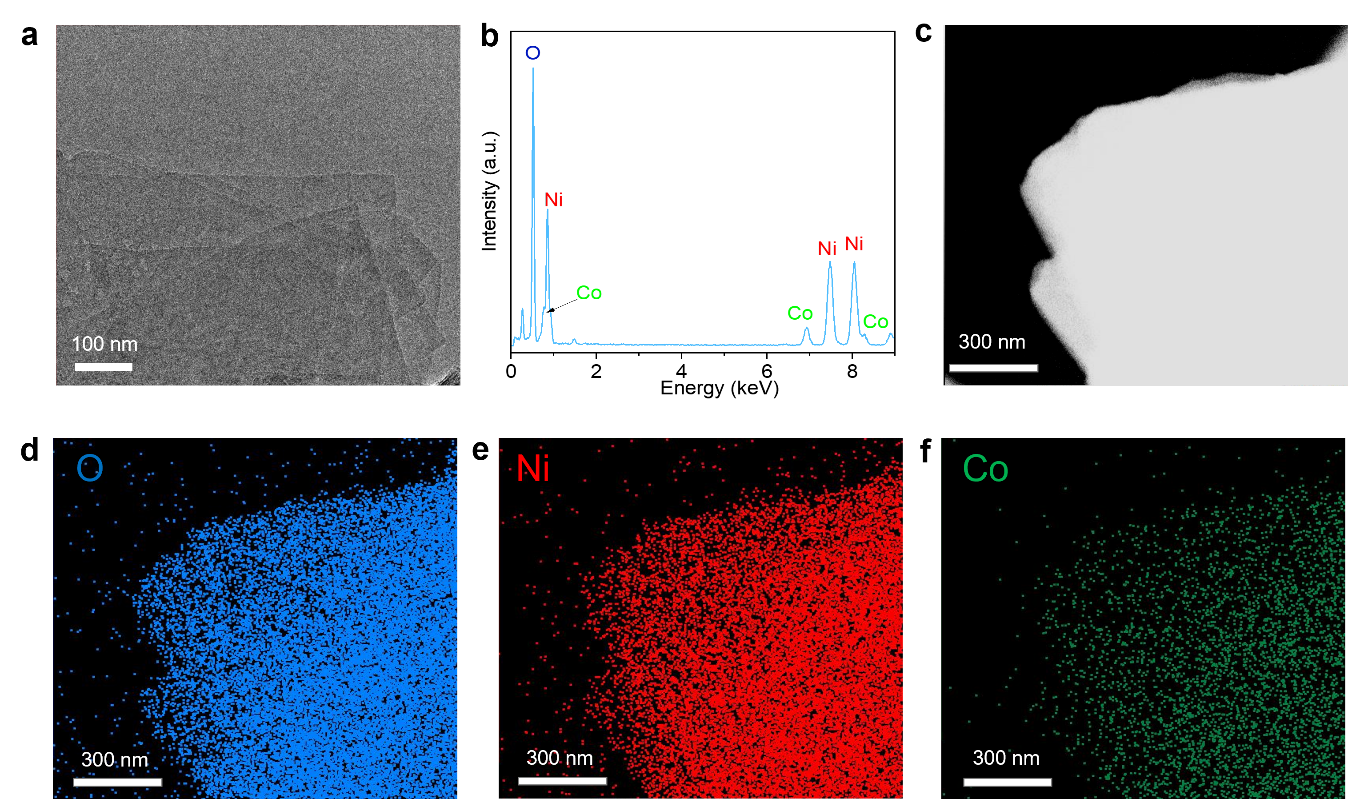


**Figure S1.** (a-b) TEM image and TEM-EDS spectrum of Ni(Co)OOH. (c-f) HAADF-TEM image and mapping images of Ni, Co and O elements in Ni(Co)OOH.


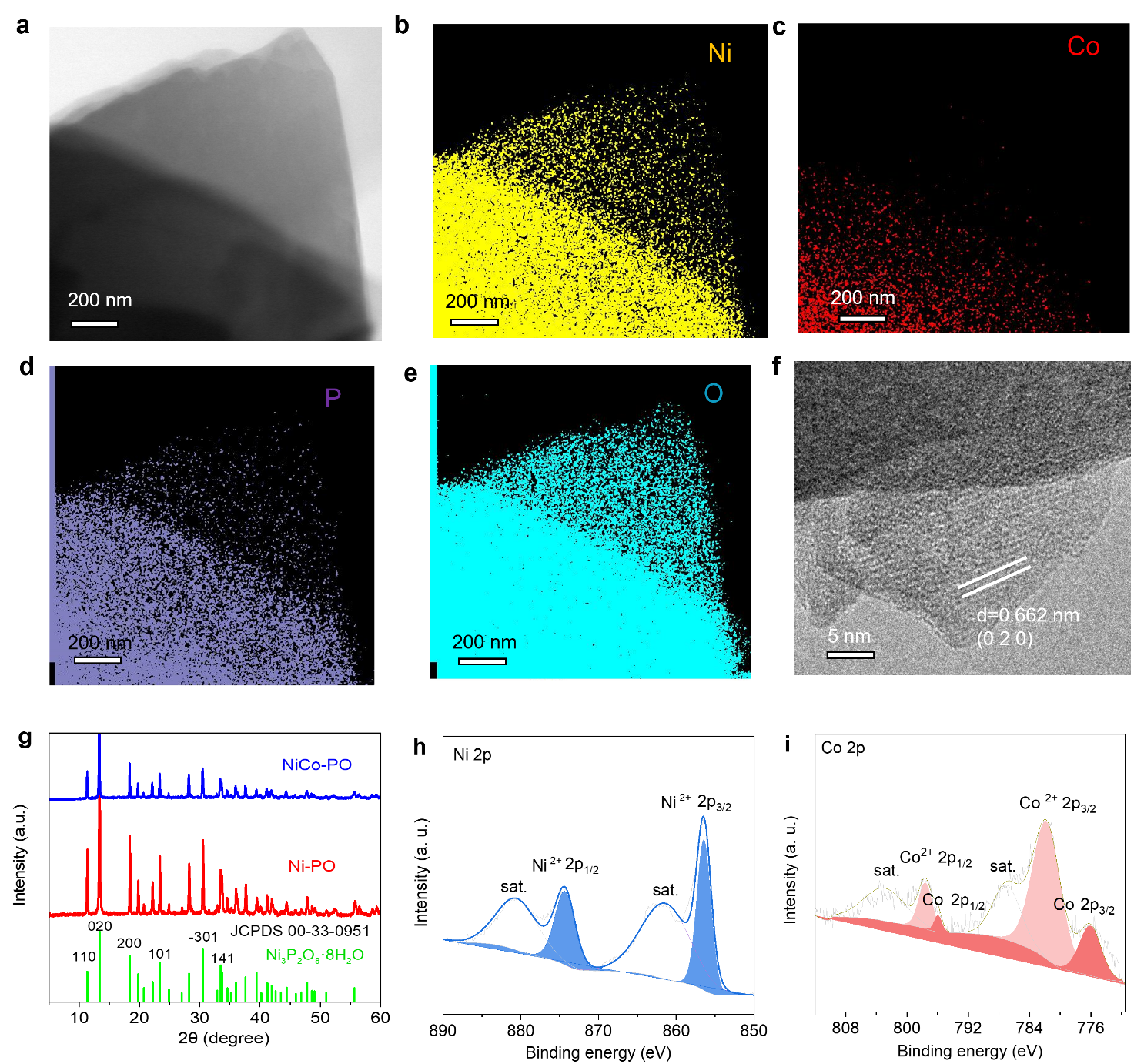


**Figure S2.** (a-f) TEM, HR-TEM and Ni, Co, P, O element mapping images of NiCo-PO. (g) XRD results of NiCo-PO and Ni-PO. (h, i) XPS spectra of Ni2p and Co2p of NiCo-PO.

From Figure S2a-e, NiCo-PO is a petal-like structure formed by stacking irregular sheet structures, and Ni, Co elements are evenly distributed. The high-resolution transmission electron microscope (HRTEM) image (Figure S2f) of NiCo-PO show the fringe spacing of 0.662 nm, corresponding to the (020) plane of the Ni_3_P_2_O_8_^.^8H_2_O. From the powder X-ray diffraction (XRD) patterns of NiCo-PO (Figure S2g), the diffraction peaks of 11.4, 13.3, 18.4, 30.6° are assigned to the (110), (020), (200), and (-301) planes of (JCPDS No. 00-33-0951), respectively, confirming the successful synthesis of the monoclinic phase. The X-ray photoelectron spectroscopy (XPS) data of NiCo-PO indicate the presence of Ni, Co, P, and O elements in NiCo-PO. In Ni2p region (Figure S2h), peaks at 856.4 eV and 874.5 eV are attributed to Ni^2+^. In Co2p region (Figure S2i), peaks at 782.0 eV and 797.6 eV are assigned to Co^2+^.


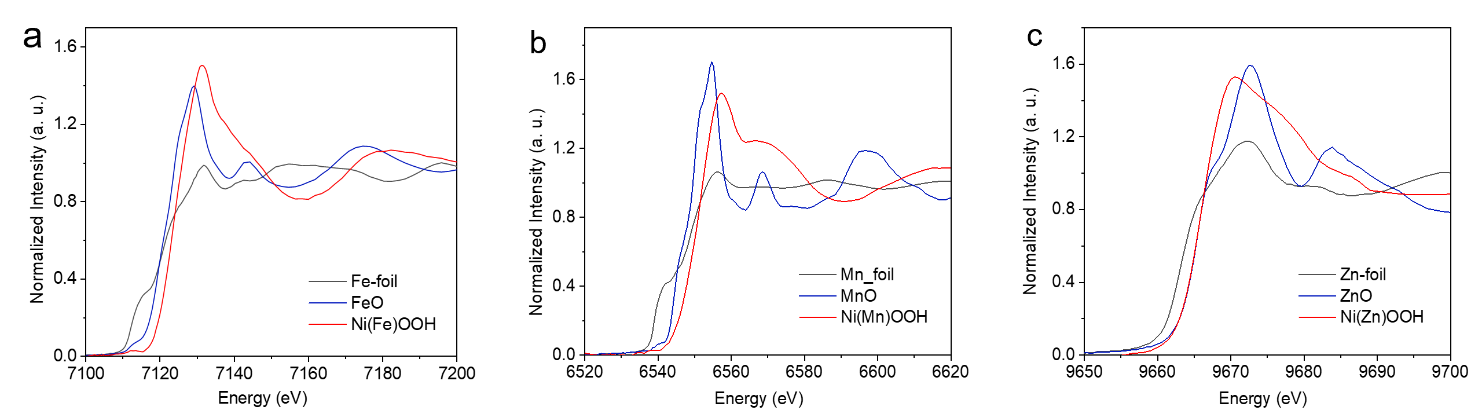


**Figure S3.** (a) Normalized XANES spectra of Fe *K*-edge for Fe foil, FeO and the Ni(Fe)OOH. (b) Normalized XANES spectra of Mn *K*-edge for Mn foil, MnO and the Ni(Mn)OOH. (c) Normalized XANES spectra of Zn *K*-edge for Zn foil, ZnO and the Ni(Zn)OOH.


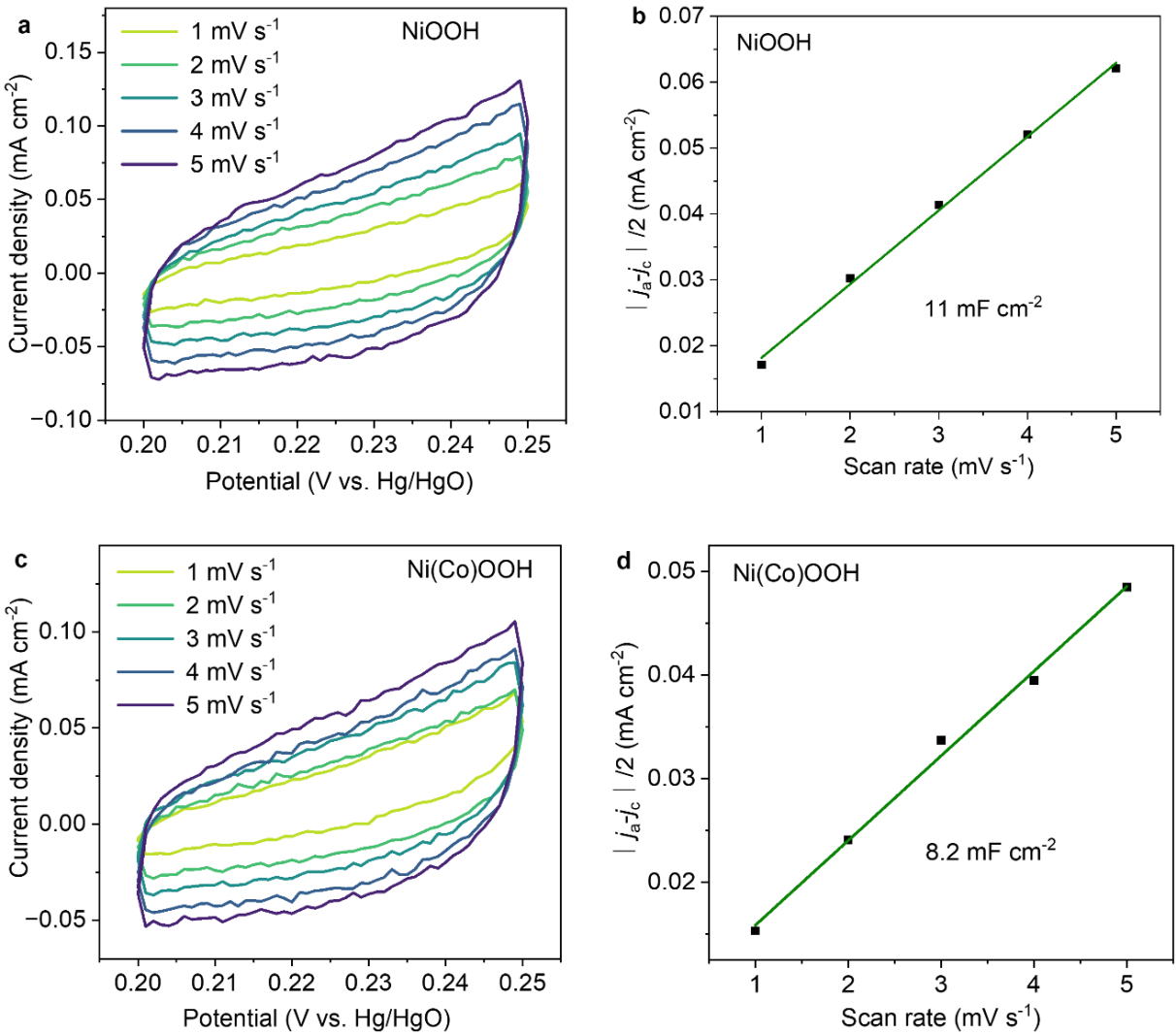


**Figure S4.** (a-b) CV curves of NiOOH catalyst at scan rates from 1 to 5 mV s^‒1^ and estimation of *C*_dl_ by plotting the capacitive current density versus the scan rate using a linear regression. (c-d) CV curves of Ni(Co)OOH catalyst at scan rates from 1 to 5 mV s^‒1^ and estimation of *C*_dl_ by plotting the capacitive current density versus the scan rate using a linear regression.


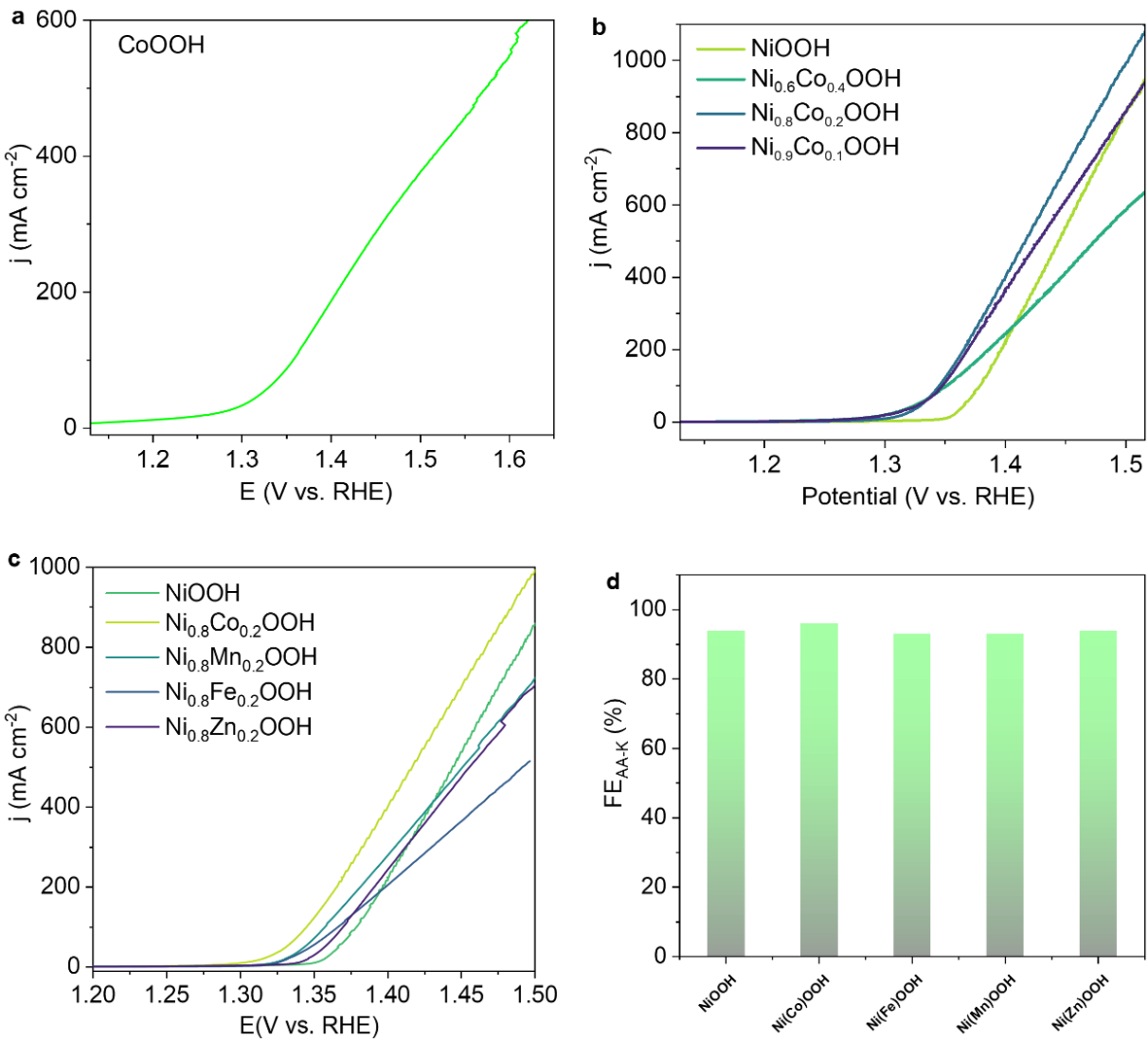


**Figure S5.** (a) LSV curve for CoOOH catalyst in 1 mol L^-1^ KOH with 0.4 mol L^-1^ LA-K solution. (b) LSV curves of the catalysts with different Ni/Co molar ratio in 1 mol L^-1^ KOH with 0.4 mol L^-1^ LA-K solution. (c) LSV Curves for NiOOH catalysts doped with different elements in 1 mol L^-1^ KOH with 0.4 mol L^-1^ LA-K solution. (d) FE_AA-K_ for NiOOH catalysts doped with different elements at 1.61 V (vs. RHE, without iR correction).


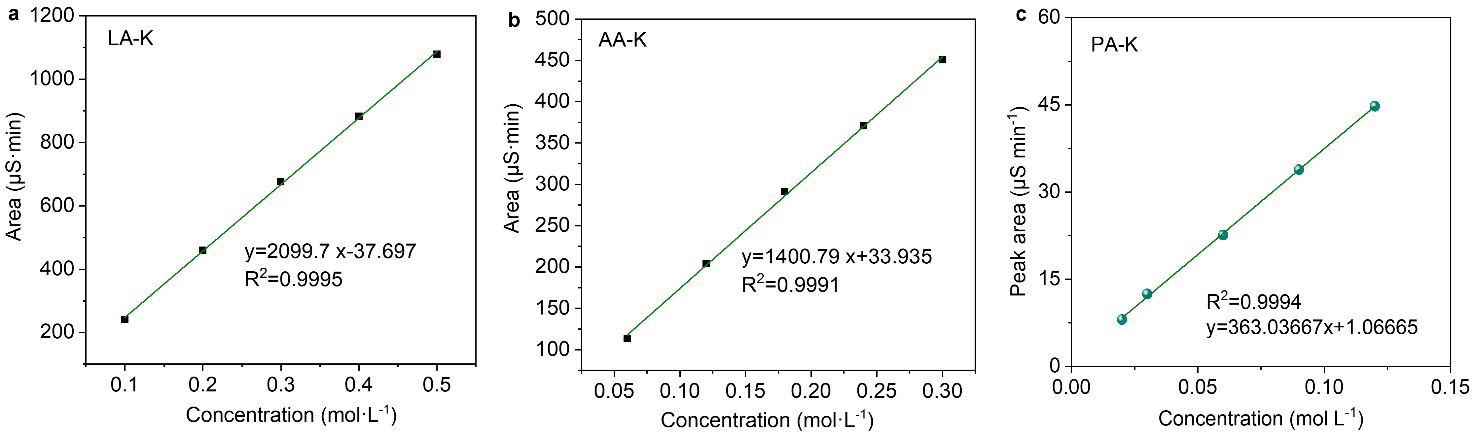


**Figure S6.** Calibration curves for (a) LA-K, (b) AA-K and (c) PA-K.


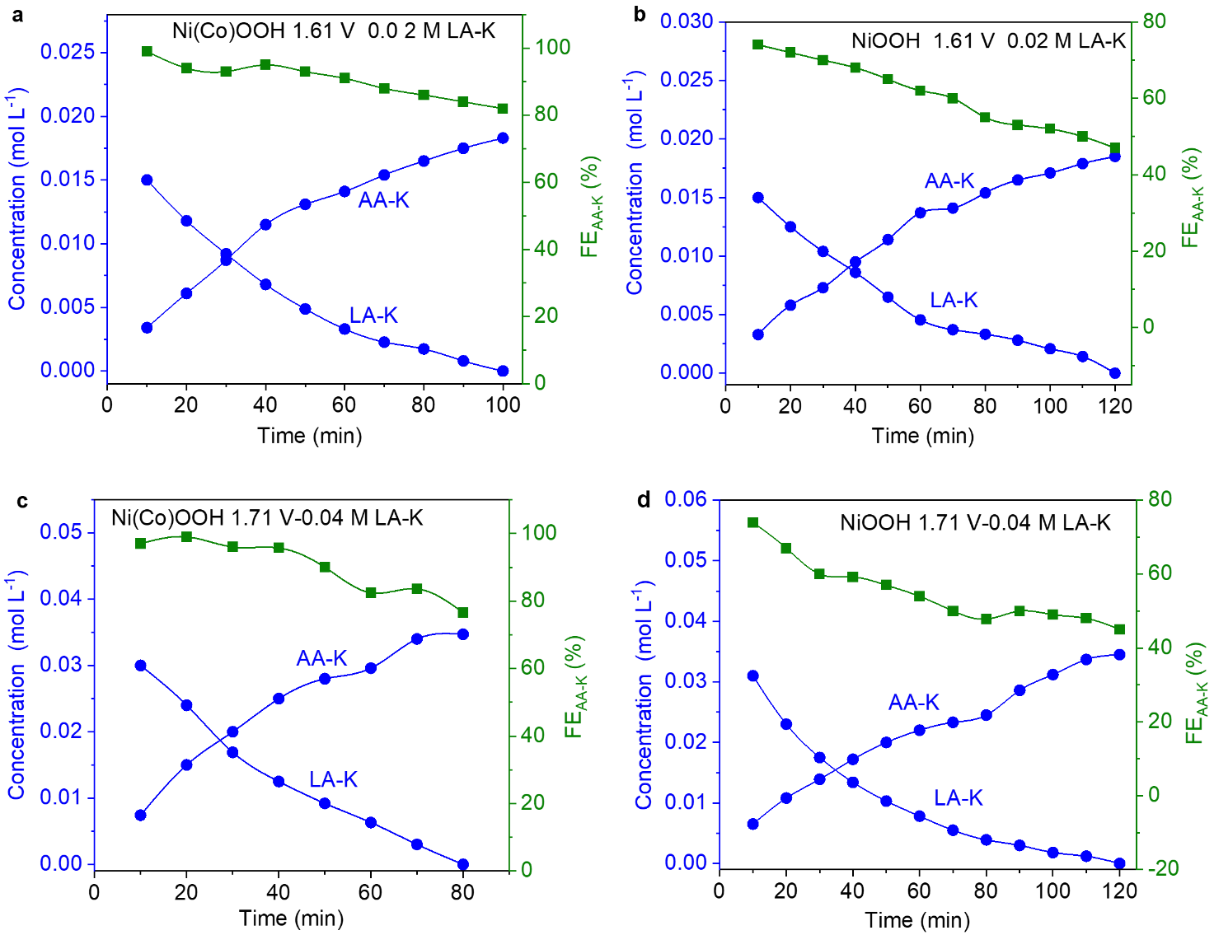


**Figure S7.** (a-b) The concentration of LA-K and AA-K, and FE_AA-K_ against time during a reaction cycle (80 mL of electrolyte with 1.0 mol L^-1^ KOH and 0.02 mol L^-1^ LA-K) for Ni(Co)OOH and NiOOH at 1.61 V (vs. RHE). (c-d) Concentration of LA-K and AA-K, and FE_AA-K_ against time during a reaction cycle (80 mL of electrolyte with 1.0 mol L^-1^ KOH and 0.04 mol L^-1^ LA-K) for Ni(Co)OOH and NiOOH at 1.71 V (vs. RHE).


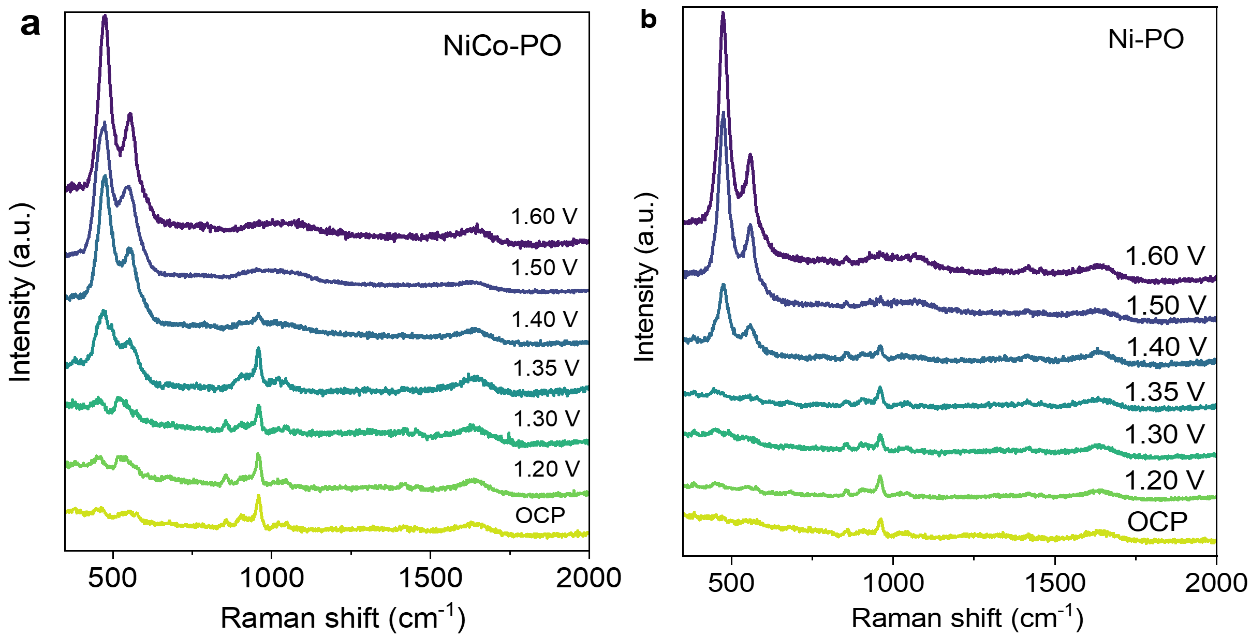


**Figure S8.** (a-b) *In situ* Raman spectra in 1.0 mol L^-1^ KOH with 0.02 mol L^-1^ LA-K at different potential for NiCo-PO and Ni-PO.


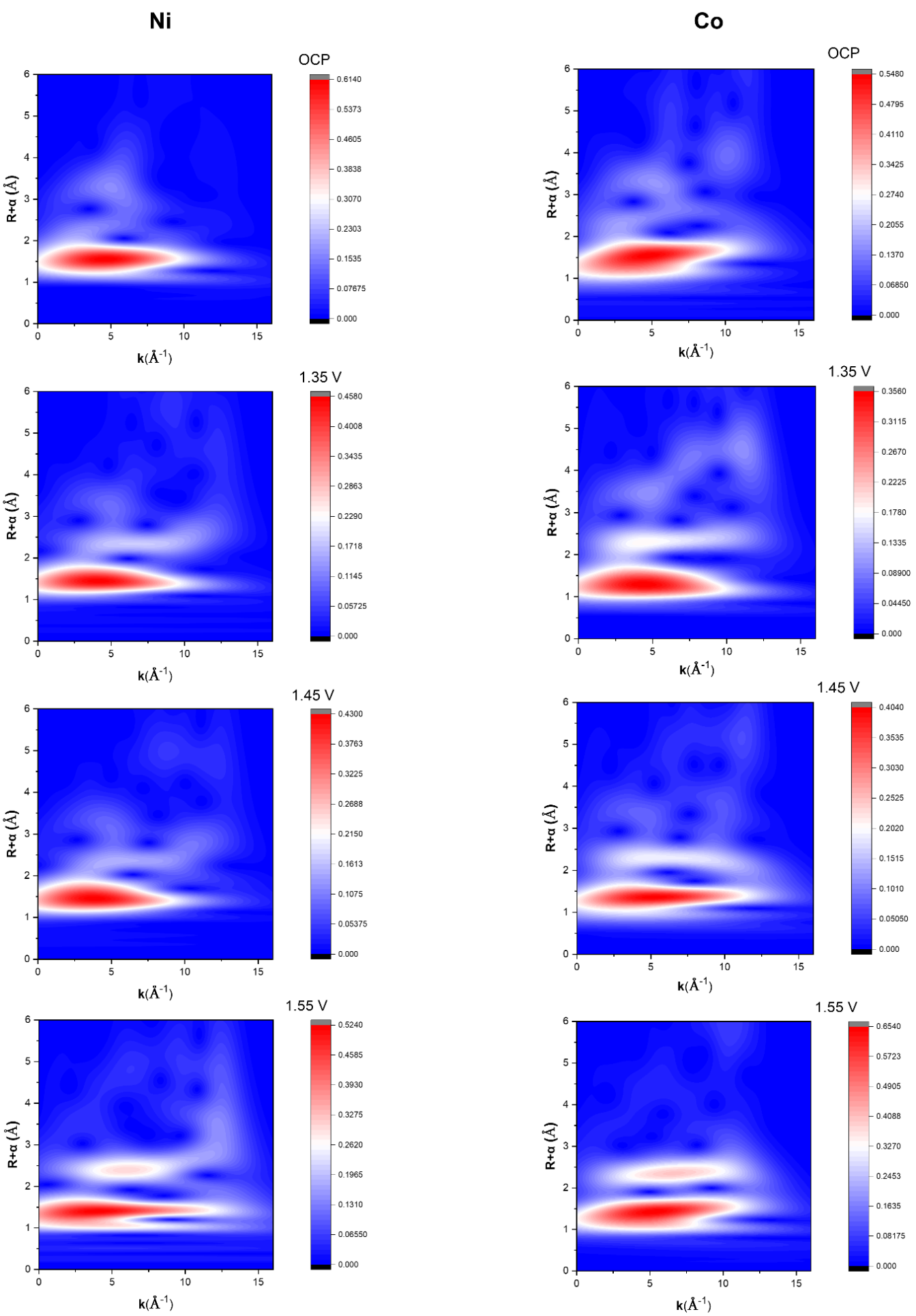


**Figure S9.** *In-situ* Ni and Co *K*-edge WT-EXAFS of Ni(Co)OOH at different potentials.


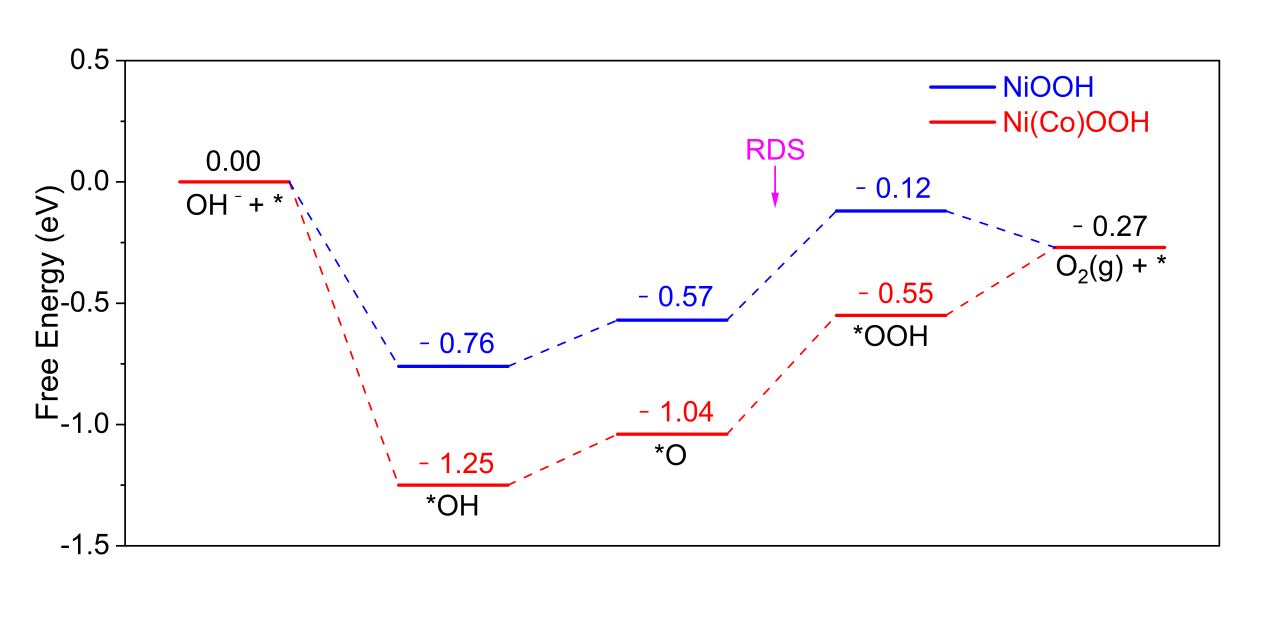


**Figure S10.** Comparison diagram illustrating the oxygen evolution reaction on NiOOH and Ni(Co)OOH catalyst (U=1.23V).


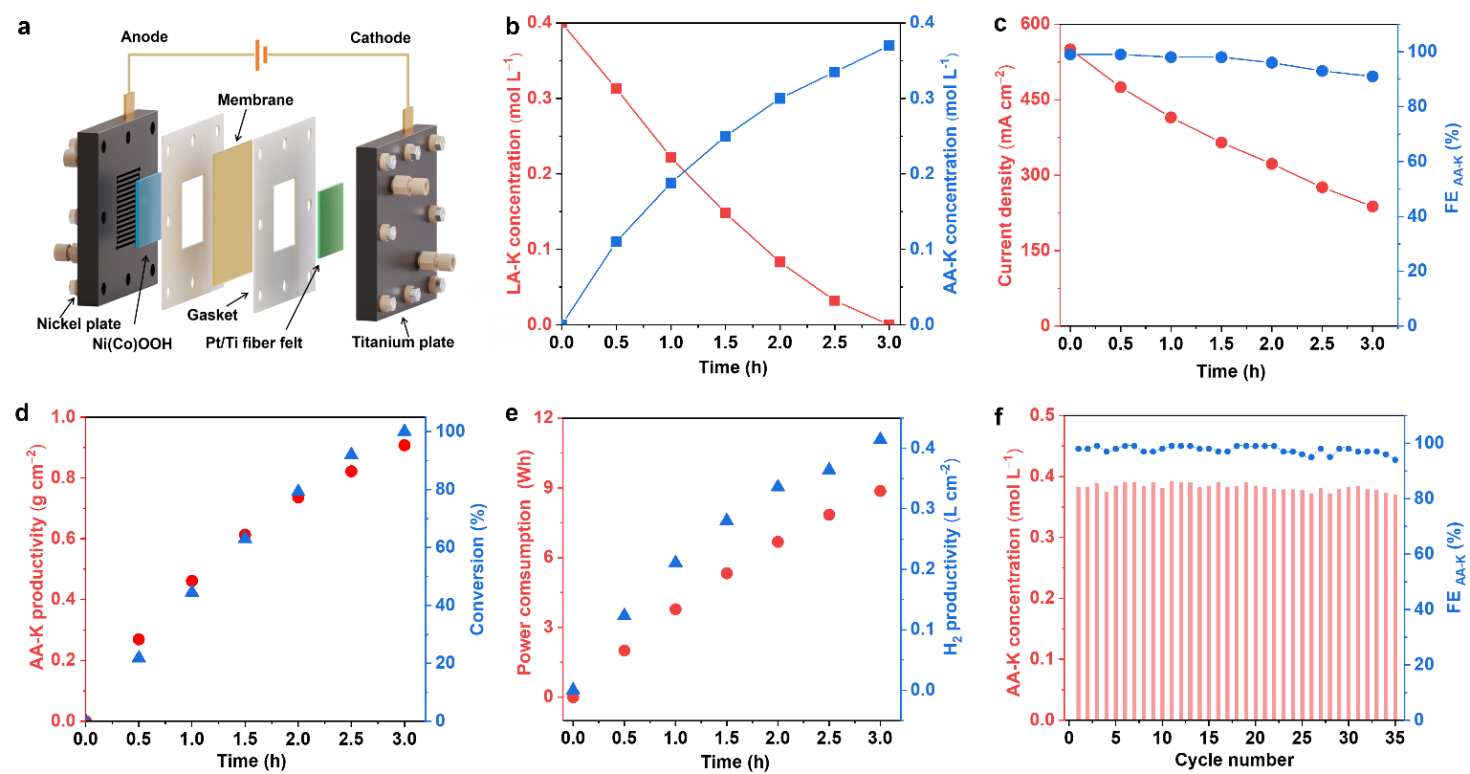


**Figure S11.** Performance of the MEA Electrolyzer. (a) Schematic diagram of the MEA electrolyzer. (b) Concentration of LA-K and AA-K as a function of time during a reaction cycle (1.0 mol L^-1^ KOH and 0.4 mol L^-1^ LA-K electrolyte). (c) Current density and FE_AA-K_ as a function of time during a reaction cycle. (d) Productivity of AA-K and conversion of LA-K as a function of time during a reaction cycle. (e) Energy consumption and H_2_ productivity as a function of time during a reaction cycle. (f) Stability evaluation for the MEA electrolyzer using Ni(Co)OOH catalyst at 2.0 V (cell voltage).

**
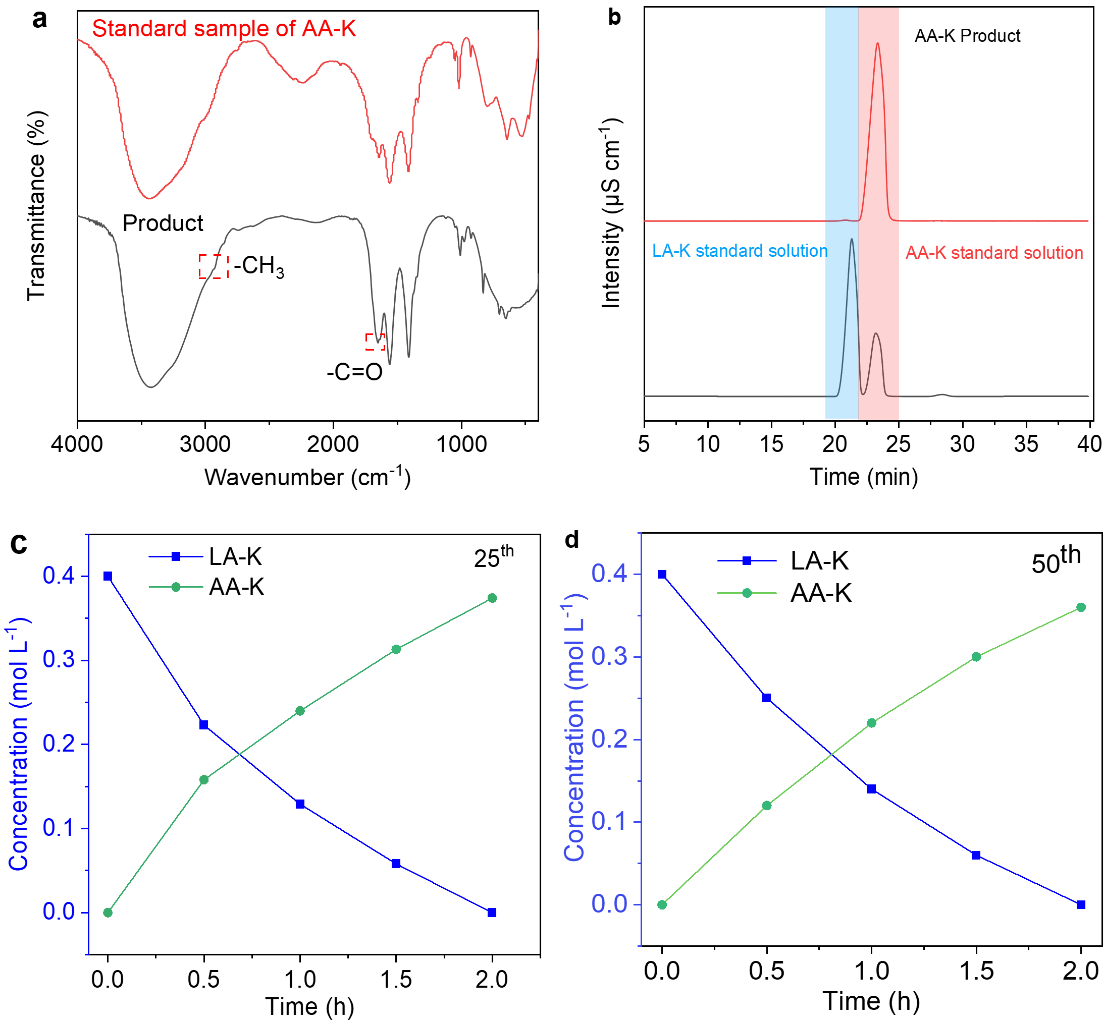
**

**Figure S12.** (a) IR spectra of the final product against time in a cycle. (b) The ion chromatography data for the AA-K product, as well as the LA-K and AA-K standard solutions. (c-d) The concentration of LA-K and AA-K against time in the 25^th^ cycle and 50^th^ cycle.

**Supplementary Table**

**Table S1.** EXAFS fitting parameters of Ni K-edge for various samples（*Ѕ*_0_^2^=0.85 from Ni-foil）*^a^CN*: coordination numbers; *^b^R*: bond distance; *^c^σ*^2^: Debye-Waller factors; *^d^* Δ*E*_0_: the inner potential correction. R factor: goodness of fit.

|  | shell | CN*^a^* | R*^b^*(Å) | σ^2^*^c^*(Å^2^) | ΔE_0_*^d^*(eV) | R factor |
| --- | --- | --- | --- | --- | --- | --- |
| Ni-foil | Ni-Ni | 12 | 2.46±0.01 | 0.0067 | 6.9±1.8 | 0.0095 |
| Ni(Co)OOH | Ni-O | 5.92±0.2 | 1.93±0.03 | 0.0082 | -4.2±2.2 | 0.0157 |
|  | Ni-O-Ni/Co | 5.98±0.3 | 2.85±0.05 | 0.0086 |  |  |

**Table S2.** EXAFS fitting parameters for the Co K-edge for various samples（*Ѕ*_0_^2^=0.75from Co-foil) *^a^CN*: coordination numbers; *^b^R*: bond distance; *^c^σ*^2^: Debye-Waller factors; *^d^* Δ*E*_0_: the inner potential correction. R factor: goodness of fit.

|  | shell | CN*^a^* | R*^b^*(Å) | σ^2^*^c^*(Å^2^) | ΔE_0_*^d^*(eV) | R factor |
| --- | --- | --- | --- | --- | --- | --- |
| Co foil | Co-Co | 12 | 2.48±0.01 | 0.0063 | 6.24±1.3 | 0.00136 |
| Ni(Co)OOH | Co-O | 5.94±0.2 | 1.90±0.01 | 0.0055 | -4.6±1.5 | 0.01705 |
|  | Co-O-Co/Ni | 5.98±0.2 | 2.82±0.02 | 0.0089 |  |  |

**Table S3.** Calculated Gibbs free energies of elementary reactions on the constructed catalyst models (1 mol·L^-1^ OH^-^, $U$ = 0 V).

| Elementary reaction | Ni(Co)OOH | |  | NiOOH | |
| --- | --- | --- | --- | --- | --- |
|  | 0 V | 1.32 V |  | 0 V | 1.32 V |
| $surface+\mathrm{LA}^{-}\to\mathrm{IM}01+\mathrm{OH}^{-}$ | -0.18 | -0.18 |  | -0.39 | -0.39 |
| $\mathrm{IM}01+\mathrm{OH}^{-}\to\mathrm{IM}02+H_{2}O+e^{-}$ | -0.66 | -1.98 |  | -0.73 | -2.05 |
| $\mathrm{IM}02+\mathrm{OH}^{-}\to\mathrm{IM}03+e^{-}$ | 1.45 | 0.13 |  | 1.51 | 0.19 |
| $\mathrm{IM}03+\mathrm{OH}^{-}\to\mathrm{IM}04+H_{2}O+\mathrm{Ac}^{-}$ | -2.28 | -2.28 |  | -2.65 | -2.65 |
| $\mathrm{IM}04+\mathrm{OH}^{-}\to\mathrm{IM}05+\mathrm{CO}_{2}+e^{-}$ | 0.14 | -1.18 |  | 0.69 | -0.63 |
| $\mathrm{IM}05+\mathrm{OH}^{-}\to surface+H_{2}O+e^{-}$ | 1.38 | 0.06 |  | 1.40 | 0.08 |
|  |  |  |  |  |  |
| $*+\mathrm{OH}^{-}\to*OH+e^{-}$ | -0.02 | -1.34 |  | 0.47 | -0.85 |
| $*OH+\mathrm{OH}^{-}\to*O+H_{2}O+e^{-}$ | 1.44 | 0.12 |  | 1.43 | 0.11 |
| $*O+\mathrm{OH}^{-}\to*OOH+e^{-}$ | 1.72 | 0.40 |  | 1.68 | 0.36 |
| $*OOH+\mathrm{OH}^{-}\to*+O_{2}+H_{2}O+e^{-}$ | 1.51 | 0.19 |  | 1.08 | -0.24 |

**Table S4.** Optimized intermediate structures on the Ni(Co)OOH, and the calculated Gibbs free energies in 1 mol·L^-1^ OH^-^.

| 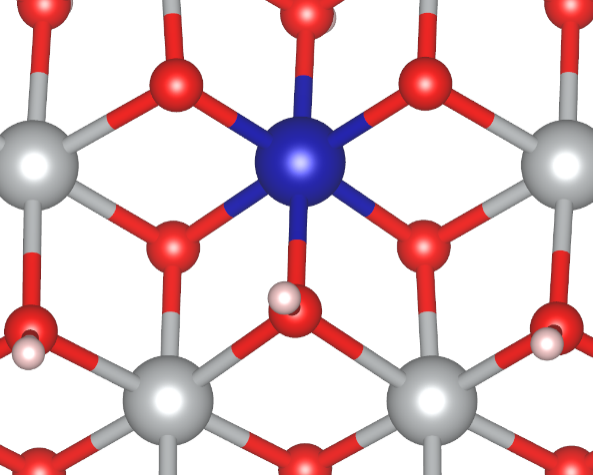Surface  -649.311731 eV | 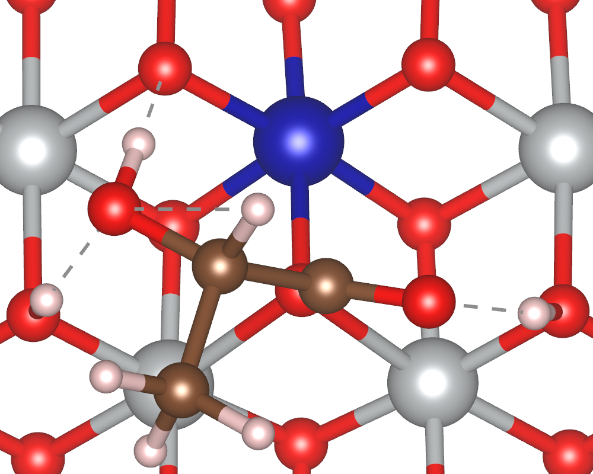IM01  -703.763747 eV |
| --- | --- |
| 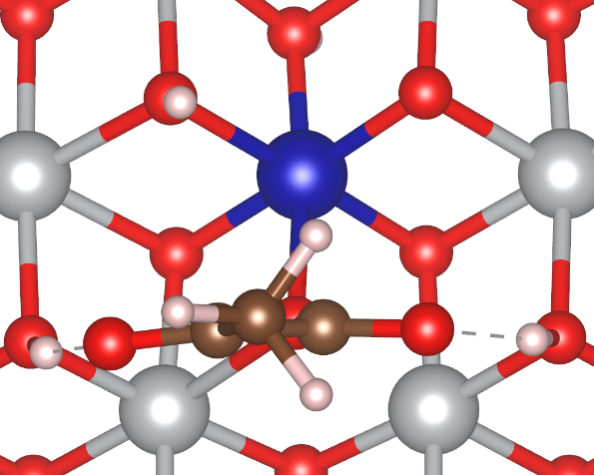IM02  -701.045943 eV | 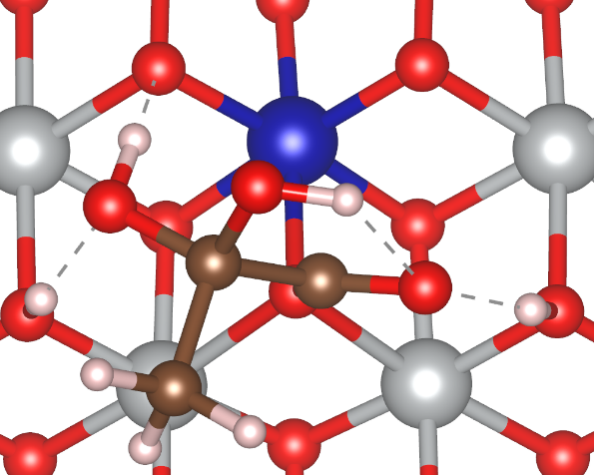  IM03  -710.302387 eV |
| 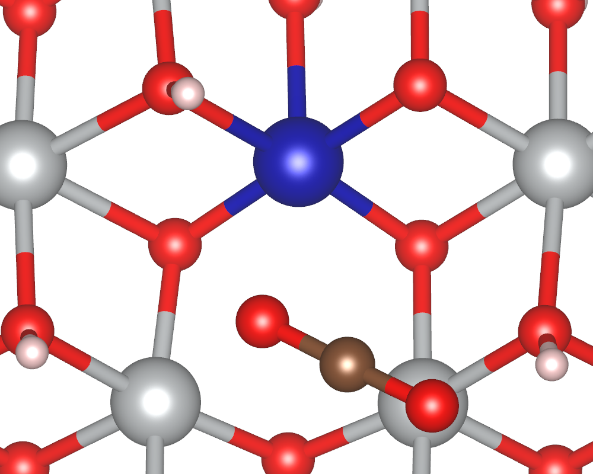IM04  -666.545982 eV | 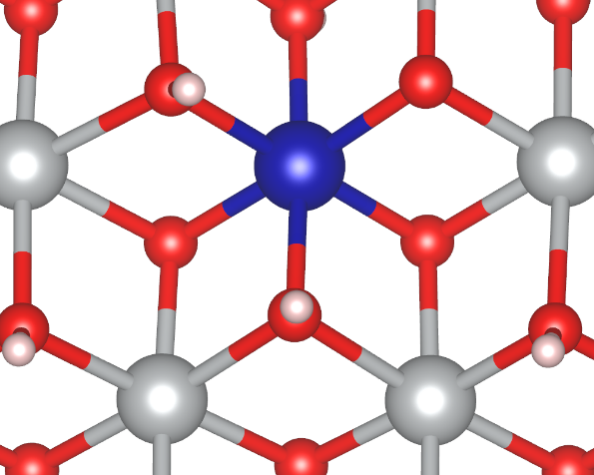  IM05  -654.067697 eV |
| 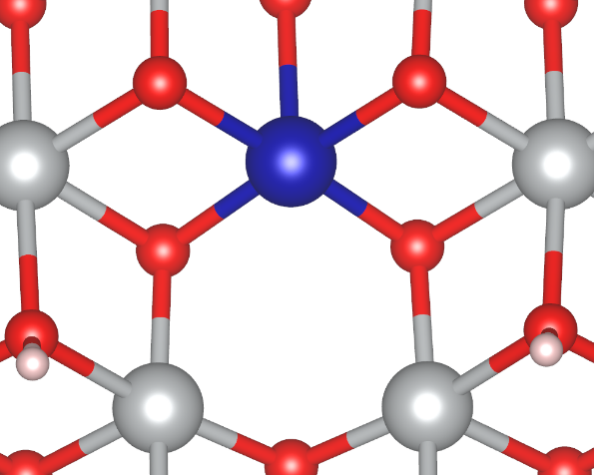  *  -638.585371 eV | 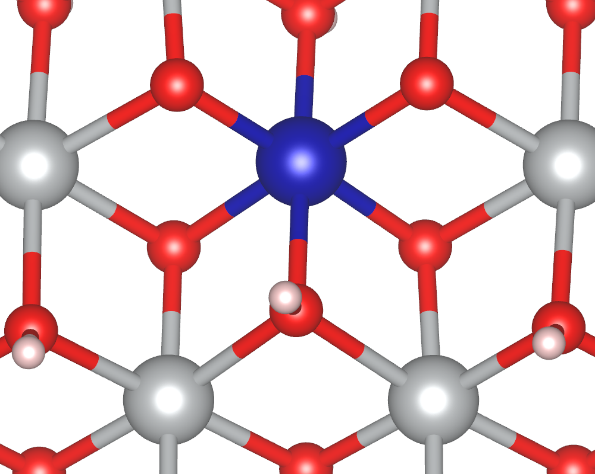*OH  -649.311731 eV |
| 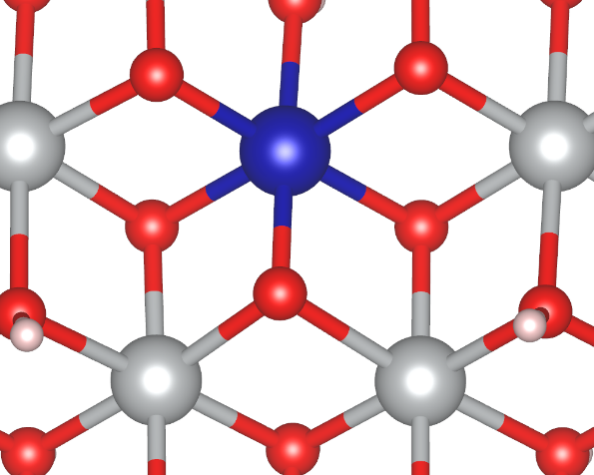  *O  -644.494621 eV | 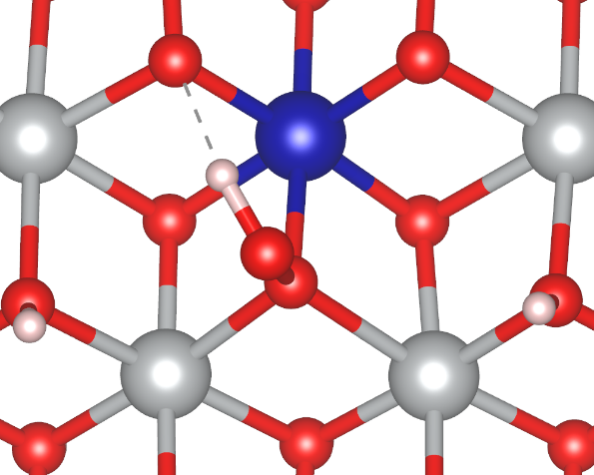  *OOH  -653.472864 eV |

**Table S5.** Optimized intermediate structures on NiOOH, and the calculated Gibbs free energies in 1 mol·L^-1^ OH^-^.

| 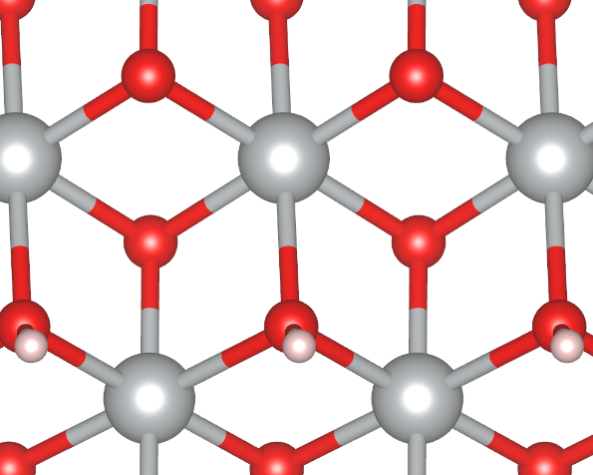  Surface  -644.396343 eV | 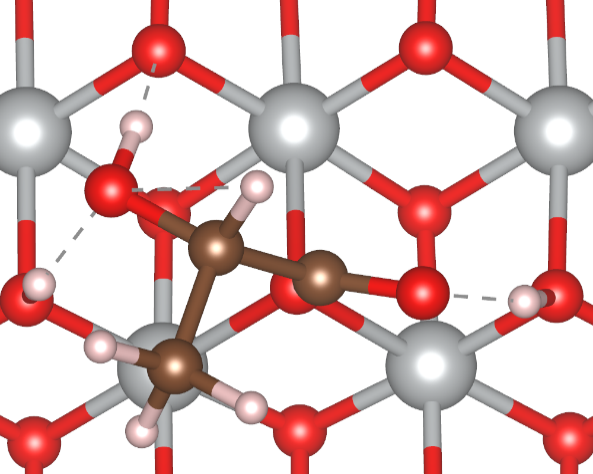  IM01  -699.054243 eV |
| --- | --- |
| 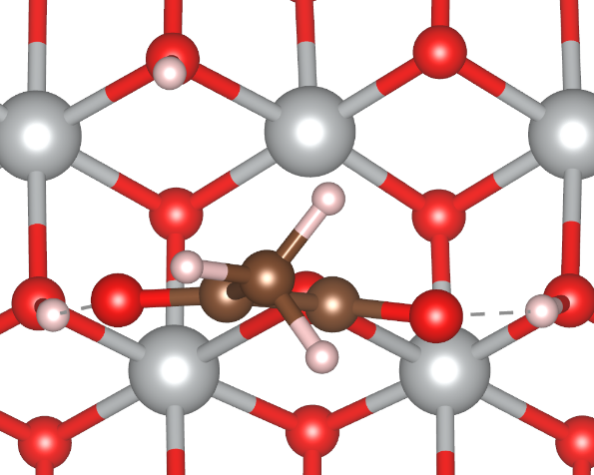  IM02  -696.410182 eV | 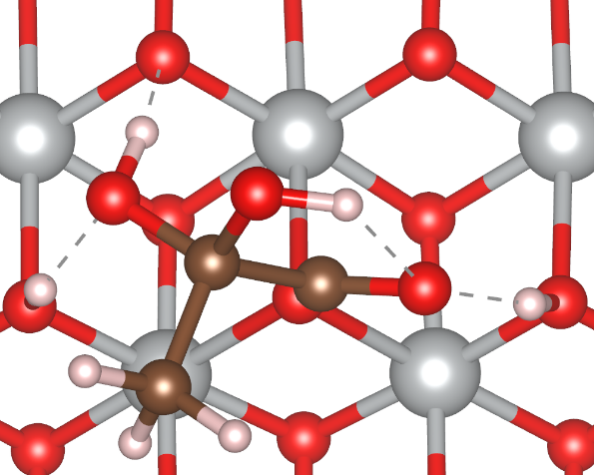  IM03  -705.600237 eV |
| 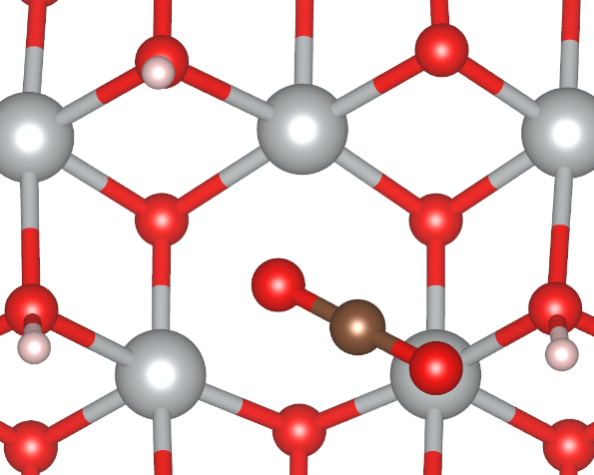  IM04  -662.212372 eV | 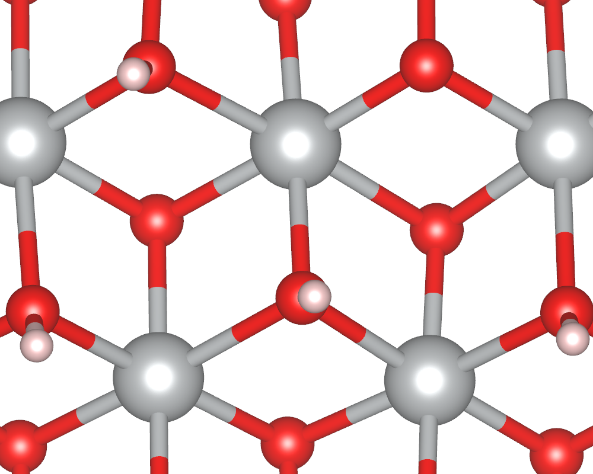IM05  -649.178530 eV |
| 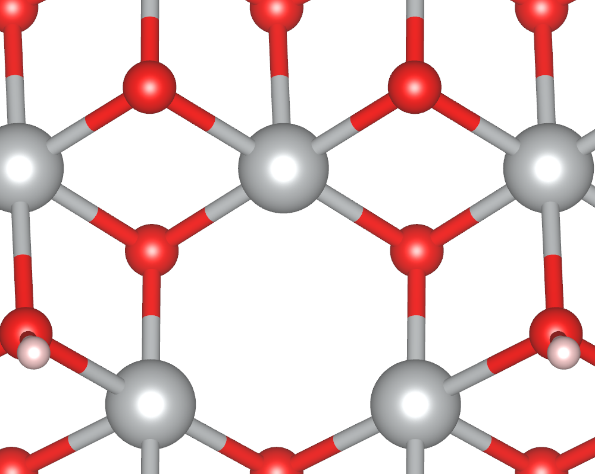  *  -634.159734 eV | 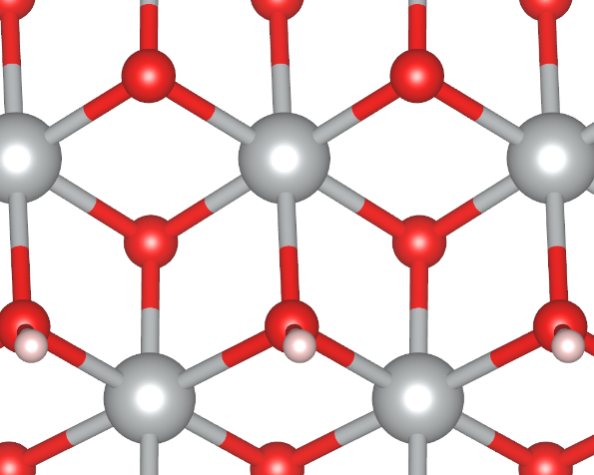  *OH  -644.396343 eV |
| 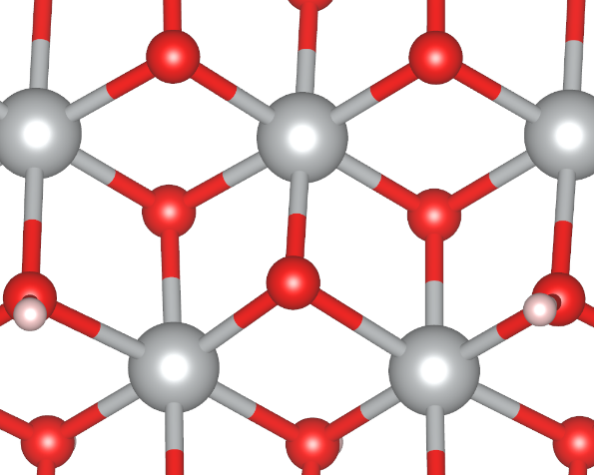*O  -639.592166 eV | 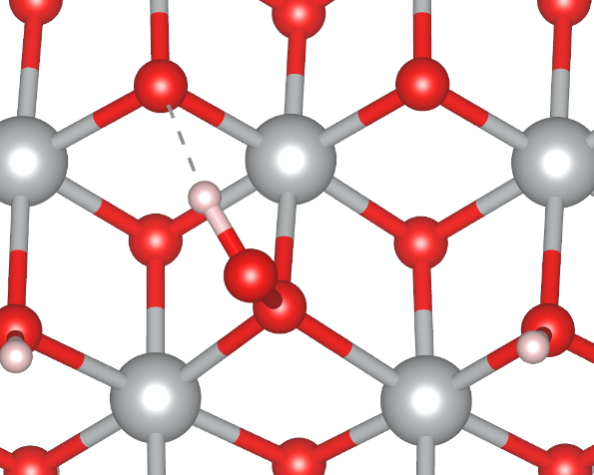*OOH  -648.618767 eV |

**Supplementary References**

[1] S. Grimme, S. Ehrlich, L. Goerigk, *J. Comput. Chem****.* 2011**, *32* (7), 1456-1465,

[2] K. Mathew, V. S. C. Kolluru, S. Mula, S. N. Steinmann, R. G. Hennig, *J. Chem. Phys.* **2019**, *151* (23), 234101.

[3] S. Ding, B. K. Xia, M. Li, F. Q. Lou, C. Cheng, T. Q. Gao, Y. X. Zhang, K. Yang, L. L. Jiang, Z. H. Nie, *Energy Environ. Sci.* **2023**, *16* (8), 3363-3372.

[4] H. Shin, K. U. Hansen, F. Jiao, *Nat. Sustainability* **2021**, *4* (10), 911-919.
